# GFAP and desmin expression in lymphatic tissues leads to difficulties in distinguishing between glial and stromal cells

**DOI:** 10.1038/s41598-021-92364-z

**Published:** 2021-06-25

**Authors:** Hauke Simon Günther, Stephan Henne, Jasmin Oehlmann, Julia Urban, Desiree Pleizier, Niclas Renevier, Christian Lohr, Clemens Wülfing

**Affiliations:** 1 Group for Interdisciplinary Neurobiology and Immunology, Biozentrum Grindel, University of Hamburg, Hamburg, Germany; 2 Division of Neurophysiology, University of Hamburg, Hamburg, Germany

**Keywords:** Biological techniques, Immunology, Neuroscience, Biomarkers

## Abstract

Recently, we found many immune cells including antigen presenting cells neurally hard wired in the T-cell zone of most lymphoid organs like amongst others, lymph nodes in rats, mice and humans. Single immune cells were reached by single neurites and enclosed with a dense neural meshwork. As it is well known that axons are always accompanied by glial cells, we were able to identify Schwann cells in the hilum, medullary and capsule region, like expected. Unexpected was the result, that we found oligodendrocyte-like cells in these regions, myelinating more than one axon. Likewise important was the finding, that one of the standard glial markers used, a polyclonal GFAP antibody equally bound to desmin and therefore marked nearly all stromal cells in cortical, paracortical and medullary cord regions. More detailed analysis showed that these results also appeared in many other non-lymphoid organs. Therefore, polyclonal GFAP antibodies are only conditionally usable for immunohistochemical analysis in peripheral tissues outside the central nervous system. It remains to be elucidated, if the binding of the GFAP antibody to desmin has its reason in a special desmin variant that can give stromal cells glial character.

## Introduction

Lymph nodes are part of both the lymphatic and the adaptive immune system and hence comprise immune cells and so-called stromal cells. In general, all cell types in lymph nodes that are of non-hematopoietic origin are classified as stromal cells^1^. Roughly, this cell population can be divided into cells with endothelial and reticular character. The former population lines all blood or lymphatic vessels and sinuses, whereas the reticular cells known as fibroblastic reticular cells (FRC) build the reticular network of collagen fibers and the conduit system^2^. The endothelial cell population includes lymphatic endothelial cells and blood endothelial cells. Lymphatic endothelial cells line the afferent and efferent lymphatic vessels, the floor of the sinuses, the walls of the trabecular sinuses, the labyrinths of lymph nodes, and the medullary sinuses. These cells also show local diversity in their expression profiles, but can generally be distinguished by expression/non-expression of CD31, LYVE-1, and podoplanin^3,4^. Blood endothelial cells line all blood vessels, including the high endothelial venules (HEV). HEV and non-HEV endothelial cells differ in their expression of CD31 and peripheral lymph node addressin (PNAd) known as MECA-79^5^.

In addition to immune cells and stromal cells, lymph nodes contain neuronal structures^6,7^ and the bi-directional communication between the nervous and the immune system is receiving more and more attention by researchers in the last decades. Beside well known humoral and endocrine pathways, there is emerging evidence for a role of hard-wired neuronal pathways, where nerve fibers directly reach cells of the immune system, especially in the parenchymal parts of lymphoid organs^8–15^. One of these neurally hard-wired connections to immune cells such as antigen presenting cells was recently described in the lymph nodes of rats, mice and humans^16,17^. Individual neurally hard-wired antigen-presenting cells (wAPCs) and other neurally hard-wired immune cells (wICs) that were contacted by single neurites were densely enclosed by a neural meshwork. While the structural relationship between neural fibers and the lymph node parenchyma is well studied, much less is known about the contribution of glial cells to the interactions between the nervous system and lymph nodes.

Schwann cells have been described as glial cells myelinating axons in the peripheral nervous system, being the counterpart to the oligodendrocytes in the central nervous system. Recently, however, researchers have described more and more additional roles Schwann cells can play^18–20^. In addition to the well-described myelinating Schwann cells, which form compact myelin layers around a single axon, it is the population of non-myelinating Schwann cells that embed or accompany multiple axons that most commonly occur in peripheral nerves^18,20^. Recently, more specialized Schwann cells have been identified in the neuromuscular junction of vertebrates. Here, the so-called perisynaptic Schwann cells are not only an essential component in developmental processes such as synaptogenesis of the neuromuscular junction, but are also involved in synaptic transmission and even synaptic remodeling after nerve injuries^21–24^. An interesting feature common to many Schwann cells is the upregulation of glial fibrillary acid protein (GFAP) expression upon activation or induced dedifferentiation^25,26^. Differentiated myelinating Schwann cells and perisynaptic Schwann cells normally do not express GFAP when contacting intact axons and neuromuscular junctions, however, in case of nerve injury, they dedifferentiate into cells with an immature Schwann cell phenotype and upregulate GFAP expression. Strong GFAP expression is also frequently found in the non-myelinating Schwann cell population^18–20,23,27,28^.

GFAP is an intermediate filament III protein not only found in the mentioned non-myelinating Schwann cells and activated myelinating Schwann cells, but also in astrocytes in the central nervous system (CNS) and in enteric glial cells^29^. It is responsible for cytoskeletal structure, maintenance of mechanical strength, and the support of neighboring neurons as well as the blood brain barrier. The GFAP protein has a head, rod and tail domain and is structurally similar to non-epithelial intermediate filaments III members such as vimentin and peripherin. This structural organization is highly conserved among all class III intermediate filament proteins^26,30,31^*.* Desmin, another type III intermediate filament, is a common marker for stromal cells of the lymph node. In addition, it is one of the earliest known muscle-specific proteins during mammalian embryonic development and is also expressed in adult skeletal and cardiac muscle progenitor cells. In contrast to the function in muscle cells, little is known about the role of desmin in endothelial or reticular cells of the lymph node stroma^4,32–35^*.*

The aim of the present study was to investigate what type of glial cells accompany axons innervating the lymph node. It turned out, that beside the detectable presence of Schwann cells in the periphery, classical glial markers detected stromal cells by false binding to desmin. Therefore, GFAP should be handled with care in lymphoid tissues.

## Materials and methods

### Organs and tissue specimens

Brain, thymus, spleen and lymph nodes (superficial cervical, facial, brachial, axillary, inguinal, popliteal) were collected from 8 weeks old female C57BL/6 J mice, Charles River Laboratories International, Inc., 251 Ballardvale Street, Wilmington, MA 01887. Organs and tissue specimens were removed post mortem and directly snap frozen in liquid nitrogen or fixed (see details in the respective section).

The organs from at least 5 animals were used for each individual staining. Right and left lymph nodes were used, respectively. All organs were cut in series and completely stained in order to obtain an overview of the signal intensity over the entire organ (same for FISH).

### Immunofluorescence and antibodies

20 µm frozen sections (Leica CM1850 cryostat / Leica Biosystems Nussloch GmbH, Nussloch, Germany) of all organ and tissue specimens were dried for 20 min. at room temperature and fixed in cold (− 20 °C) Acetone Methanol (1:1). Blocking of unspecific binding sites was performed by a 5 min. incubation (room temperature) with Super-Block (ScyTek Laboratories, Logan, Utah, USA) and a subsequent washing step for 5 min. in PBS (phosphate buffered saline).

Primary antibodies used: rabbit anti-MBP (myelin basic protein/polyclonal), (1:100; Abcam plc, Cambridge, UK/ order No. ab40390), rabbit anti-GFAP (monoclonal), (1:500; Abcam plc, Cambridge, UK/order No. ab33922), rabbit anti-S100B (monoclonal), (1:100; Abcam plc, Cambridge, UK/order No. ab52642), rabbit anti-GFAP (polyclonal) (1:100; DAKO [Agilent], Santa Clara, USA/order No. Z0334), rabbit anti-LYVE-1 (polyclonal) (1:100; Abcam plc, Cambridge, UK/ order No. ab14917), mouse anti-NF (SMI312) (1:100; BioLegend, San Diego, USA/order No. SMI-312R-500), hamster anti-podoplanin (monoclonal) (1:600; Abcam plc, Cambridge, UK/order No. ab11936), rabbit anti-Collagen III (polyclonal) (1:600; Abcam plc, Cambridge, UK/order No. ab7778), rabbit anti-CD31 (polyclonal) (1:50; Abcam plc, Cambridge, UK / order No. ab28364), mouse anti-desmin (monoclonal) (1:50; Abcam plc, Cambridge, UK/ order No. ab8470), rabbit anti-OligoSpecific/claudin 11 (polyclonal) (1:100; Abcam plc, Cambridge, UK/ order No. ab53041).

Primary antibodies were diluted in PBS as described above and incubated for 60 min. up to 120 min. at room temperature or overnight at 4 °C, followed by 15 min. incubation of secondary antibodies.

Secondary antibodies used goat anti-rabbit-DyLight 549 (1:700; Biomol, Hamburg, Germany), goat anti-rabbit-Alexa 488 (1:700; Biomol, Hamburg, Germany), goat anti-hamster-Alexa 488 (1:000; Abcam plc, Cambridge, UK) goat anti-mouse-DyLight 549 (1:700; Biomol, Hamburg, Germany) and goat anti-mouse-Alexa 488 (1:700; Biomol, Hamburg, Germany).

For multicolor staining, primary antibodies in certain cases were conjugated with: Cy3 (Cy3 Fast Conjugation Kit/Abcam plc, Cambridge, UK), FITC (FITC Fast Conjugation Kit/Abcam plc, Cambridge, UK), HiLyteFluor 647 (AnaTag HiLyte Fluor 647/ANASPEC, Inc., Fremont, USA) and HiLyteFluor 488 (AnaTag HiLyte Fluor 488 / ANASPEC, Inc., Fremont, USA).

Slides were mounted using Vectashield hard set mounting medium with DAPI (Vector Laboratories, Burlingame, CA, USA).

The establishment of the antibody staining was carried out on the respective positive tissue. GFAP, MBP, S100B and neurofilament were established on brain (for examples, see supplementary Fig. 7), all other markers were established on cervical lymph nodes. Each antibody staining was accompanied by a negative control in which only secondary antibodies were used*.*

A Zeiss Axio Imager.M2m microscope (Carl Zeiss, Oberkochen, Germany) with Zen software was used for image collection.

### Silver staining

The silver staining protocol was taken from the publication of Novotny^36^. Briefly, the following steps were conducted:

PFA fixed, paraffin embedded sections are dewaxed and hydrated in distilled water via descending alcohols. Pretreated samples were incubated with 30% silver nitrate overnight, reduced in reducing solution, incubated in ammoniacal silver nitrate for 15 min., reduced second time and incubated for 5 min. in 5% sodium thiosulfate. After dehydrating via ascending alcohols, terpineol-xylene the samples were embedded in DePeX.

### Western blot

All organs and tissue specimens (fresh) were homogenized with a single cell suspension kit (Minute Single Cell Isolation Kit, Invent Biotechnologies Plymouth, MN 55447, USA, Protocol A). Used Lysis-Buffer contains: 125 mM Tris/5 mM EDTA/200 mM NaCl/0.2% SDS (pH 8). Protein samples were prepared and applied to SDS-PAGE and transferred to a nylon filter. Western blot was performed using anti-GFAP (rabbit anti-GFAP (monoclonal), (Abcam plc, Cambridge, UK/ order No. ab33922), rabbit anti-GFAP (polyclonal) (DAKO [Agilent], Santa Clara, USA/order No. Z0334)) and anti-desmin antibodies (mouse anti-desmin (monoclonal) (Abcam plc, Cambridge, UK/order No. ab8470)).

Organs from 3 animals each were used for protein analyzes. The lymph nodes were pooled per animal.

### Fluorescence in situ hybridization

For the investigation of the GFAP mRNA transcript we used ready-to-use probes from Biosearch Technologies. Tissue (lymph node and brain [control]) were snap frozen in liquid nitrogen and processed to 20 µm frozen sections (Leica CM1850 cryostat/Leica Biosystems Nussloch GmbH, Nussloch, Germany). Hybridization was carried out according to the standardized protocol for the probe “Stellaris FISH Probes, Mouse GFAP with Quasar 570 Dye” and finally evaluated microscopically.

### Electron microscopy

All samples were either fixed in 2% buffered glutaraldehyde (GA) or a mixture of 2% paraformaldehyde (PFA) and 0.1% GA in 0.05 M MSB pH 6.8 (microtubule stabilizing buffer containing: Pipes (100 mM) EGTA (10 mM) and MgSO_4_ (5 mM).

### Transmission electron microscopy (TEM)

For TEM studies, samples were embedded in LR White resin (Merck KGaA, Darmstadt, Germany) and cut to a thickness of 70 nm on a Reichert-Jung UltraCut E microtome. Immunogold staining was performed post embedding using the polyclonal primary anti-GFAP antibody (DAKO) 1:200 in MSB for 2 h at room temperature. A colloidal gold 18 nm secondary antibody was used diluted 1:50 in MSB with incubation time of 2 h at room temperature. Subsequently, samples were post fixed in 1% GA and incubated in 1% aqueous Uranyl acetate and 1% lead nitrate respectively for an overall enhanced tissue contrast. Imaging was performed on a transmission electron microscope Zeiss EM 902A equipped with a wide-angle dual speed 2 K-CCD-Camera at 80 kV acceleration voltage.

#### Scanning electron microscopy (SEM)

After fixation samples were embedded in low melting point agarose (Merck KGaA, Darmstadt, Germany) and cut to a thickness of 80 µm on a vibratome (Leica VT 1000 S) using a sapphire blade (Plano GmbH, Wetzlar, Germany). Immunohistochemistry was conducted with the polyclonal anti-GFAP (DAKO) primary antibody as described above in combination with a 549 nm FluoroNanogold secondary antibody (Nanoprobes, Inc. NY, USA). Samples were post fixed in 1% GA and enhancement of gold particles was conducted using the GoldEnhance Kit (Nanoprobes) with a development time of 2–5 min resulting in a particle size of about 20 – 30 nm. Subsequently, tissue was dehydrated with ascending alcohols and finally critical point dried with carbon dioxide as intermediate medium (Leica CPD 300 critical point dryer). Samples were mounted on conductive tape and sputter-coated with carbonite (Leica ACE 600 carbon coater). Imaging was performed on a scanning electron microscope LEO 1525 (Carl Zeiss Microscopy GmbH, Jena, Germany) at 15–20 kV acceleration voltage.

#### Sample preparation for 2DE electrophoresis

Sample preparation was performed according to Proteome Factory’s 2DE sample preparation protocol for tissue (snap frozen samples pooled from 2 animals). Six volumes of preparation buffer [containing 9 M urea, 70 mM DTT, 2% ampholytes 2–4] was added sample after disruption of tissue using mortar and pistil at -190 °C (cooling by liquid nitrogen). Next the sample was five times frozen under liquid nitrogen and thawed followed by incubation for 30 min at room temperature and centrifugation for 45 min at 15,000×g. The supernatant was removed and frozen in new tubes at -80 °C.

#### Two dimensional gel electrophoresis, blotting, immunostaining

Two dimensional gel electrophoresis (2DE) was performed according to Proteome Factory’s 2D electrophoresis technique. 130 µg of protein was applied to vertical rod gels (9 M urea, 4% acrylamide, 0.3% PDA, 5% glycerol, 0.06% TEMED and 2% carrier ampholytes (pH 2–11), 0.02% APS) for isoelectric focusing at 8820 Vh in the first dimension. After focusing, the IEF gels were incubated in equilibration buffer, containing 125 mM trisphosphate (pH 6.8), 40% glycerol, 65 mM DTT, and 3% SDS for 10 min and subsequently frozen at − 80 °C. The second dimension SDS-PAGE gels (20 cm × 30 cm × 0.1 cm) were prepared, containing 375 mM Tris–HCl buffer (pH 8.8), 12% acrylamide, 0.2% bisacrylamide, 0.1% SDS and 0.03% TEMED. After thawing, the equilibrated IEF gels were immediately applied to SDS-PAGE gels. Electrophoresis was performed using 140 mA for 5 h until the front reached the end of the gel.

One gel was stained with FireSilver (Proteome Factory, Berlin, Germany) for preparative applications; the other gel was used for immunoblotting. 2DE gels were blotted using an Immobilon-P membrane (PVDF, pore size 0.45 mm; Millipore, Bedford, USA) and a Trans-Blot SD Semi-Dry Transfer Cell (Biorad, Munich, Germany) at a constant current and 5 V over night at 4 °C. After washing and blocking, membranes were incubated with anti-GFAP-AB (diluted 1:500 in TBS Tween containing 1% (w/v) BSA) over night and then incubated with peroxidase conjugated anti-rabbit IgG (Sigma, Taufkirchen, Germany, diluted 1:2500 in TBS Tween containing 1% (w/v) BSA) for 2 h. Immunoblots were developed with TMB (Seramun, S-709-1-TMB). Between all incubation steps the membrane was washed with TBS Tween (5 times for 10 min).

#### nanoHPLC-ESI–MS/MS

Protein identification was performed by Proteome Factory (Proteome Factory AG, Berlin, Germany). Immune positive protein spots were in-gel digested by trypsin (Promega, Mannheim, Germany) and analysed by nanoHPLC-ESI–MS/MS. The LCMS system consisted of an Agilent 1100 nanoHPLC system (Agilent, Waldbronn, Germany), PicoTip electrospray emitter (New Objective, Woburn, MA) and an Orbitrap XL or LTQ-FT Ultra mass spectrometer (ThermoFisher Scientific, Bremen, Germany). Peptides were first trapped and desalted on the enrichment column (Zorbax 300SB-C18, 0.3 × 5 mm, Agilent) for five minutes (solvent: 2.5% acetonitrile/0.5% formic acid), then separated on a Zorbax 300SB-C18, 75 µm × 150 mm column (Agilent) using a linear gradient from 10 to 32% B (solvent A: 5% acetonitrile in water, solvent B: acetonitrile, both with 0.1% formic acid). Ions of interest were data-dependently subjected to MS/MS according to the expected charge state distribution of peptide ions. Proteins were identified by database search against the Swissprot protein database (European Bioinformatics Institute (EMBL-EBI), Hinxton Cambridge, UK) using MS/MS ion search of the Mascot search engine (Matrix Science, London, England). Peptide mass tolerance was ± 3 ppm, fragment mass tolerance was ± 0.6 Da. Carbamidomethylation of cysteine was assigned as fixed while Deamidation (NQ) and Oxidation (M) were selected as variable modifications.

## Results

### Detection of glial cells

**Figure 1:**
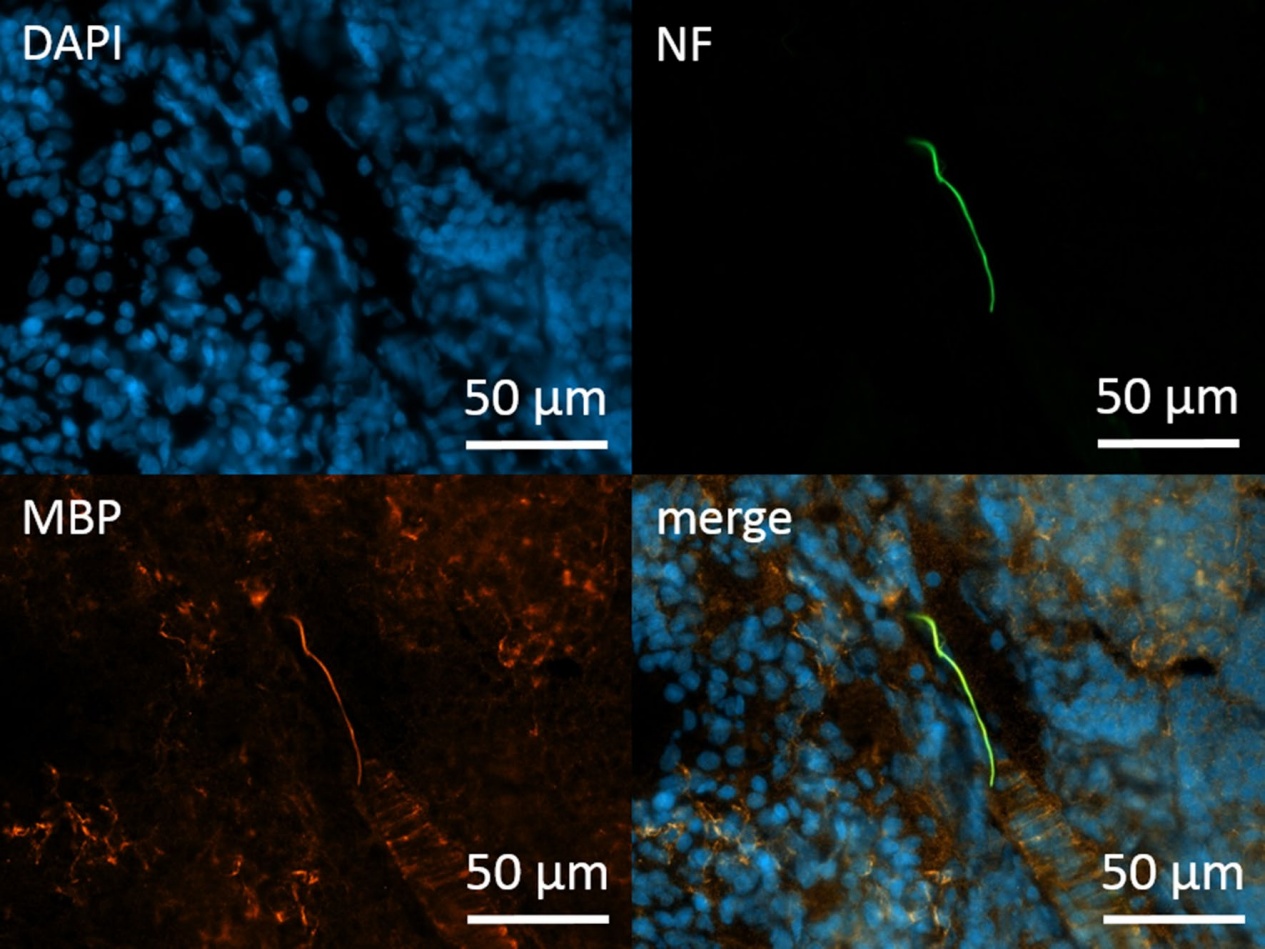
Immunhistochemical staining for Schwann cells in the medulla of a cervical lymph node. Co-staining of neurofilament SMI 312 (directly conjugated, green) and myelin basic protein (MBP) (orange). DAPI for nuclear staining in blue.

In the first part of this work, we wanted to understand which types of peripheral glia cells accompany the nerves that enter the lymph node and form the recently describes neural structures such as the wIC, wAPC and the neural nexus system. We used antibodies against myelin basic protein (MBP) for staining myelinating Schwann cells. We sparsely detected positive staining for MBP in the lymph node mainly located in the hilum, the medulla and in the area of the capsule (Fig. 1). For detection of non-myelinating Schwann cells, we decided to use S100B and GFAP. We detected S100B positive staining in the hilum, the medulla and in the area of the capsule, but not in other parts of the lymph node (supplementary Fig. 1).

**Figure 2:**
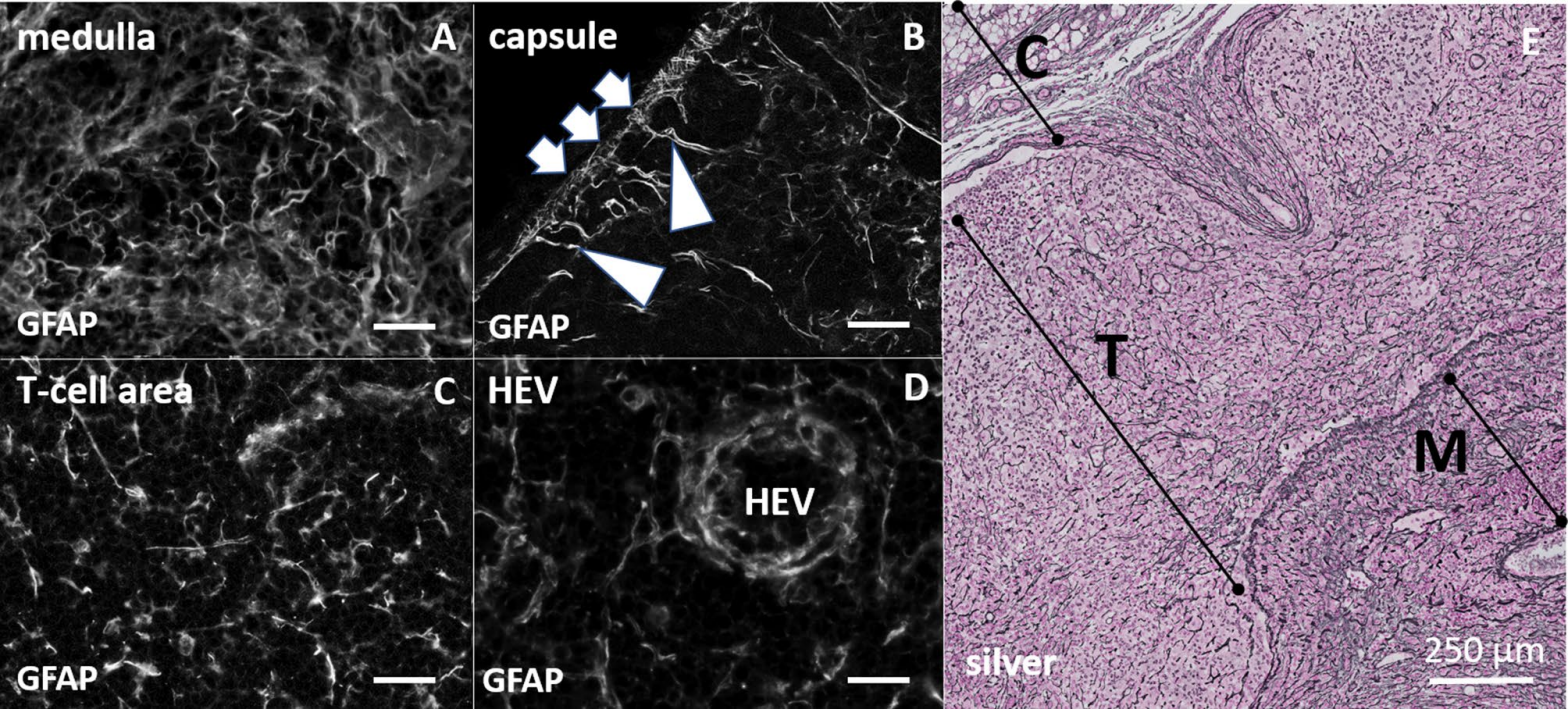
Left site (**A**–**D**), immunohistochemical staining with polyclonal anti-GFAP antibody (DAKO) in different areas of the lymph node, colorless display for impartial interpretation. (**A**) Undirected arrangement of branched structures in the medulla. (**B**) Clear signals in the capsule (arrow). Radially symmetric inward extending of longer directional signals (arrowheads). (**C**) Heterogeneous signal distribution with clearly reticular arrangement of signals in the T-cell area. (**D**) Concentric and dense packed signals around the high endothelial venules (HEV) (Scale bar for **A**–**D** 50 µm). Right site (**E**) silver staining of lymph node cross section. (**E**) Digital enlargement of a silver-stained cross-section through a lymph node. Magnification covers as much as possible areas from the capsule (C), T-cell region (T) and the medulla (M). The fibrous structures of this staining represent the reticular network in the lymph node.

For staining glial fibrillary acid protein, we used a frequently employed polyclonal GFAP antibody from DAKO (see Methods). GFAP immunoreactivity was distributed throughout the lymph node, with a filamentary phenotype showing different densities depending on the lymph node micro-domain (Fig. 2A–D). GFAP immunoreactivity resembled a classical silver staining of reticular fibers (Fig. 2E). Anti-GFAP staining was present in the capsule (Fig. 2B), coursed through the sinuses, lined the subsinoidal layer and formed the same network-like structures as collagen fibers do in cortical, paracortical or medullary areas. Furthermore, the thick and undulating GFAP-positive structures in the medulla (Fig. 2A) differed in their appearance from the much finer and straighter structures in the cortex, in particular in the T-cell zone (Fig. 2C)*.* They also surrounded high endothelial venules (HEV), again resembling the distribution seen with reticular fibers in a silver staining, which shows well described channels around the entry and exit sites for lymphocytes (Fig. 2D)*.*

### Detection of stromal cells with the glial marker GFAP

**Figure 3:**
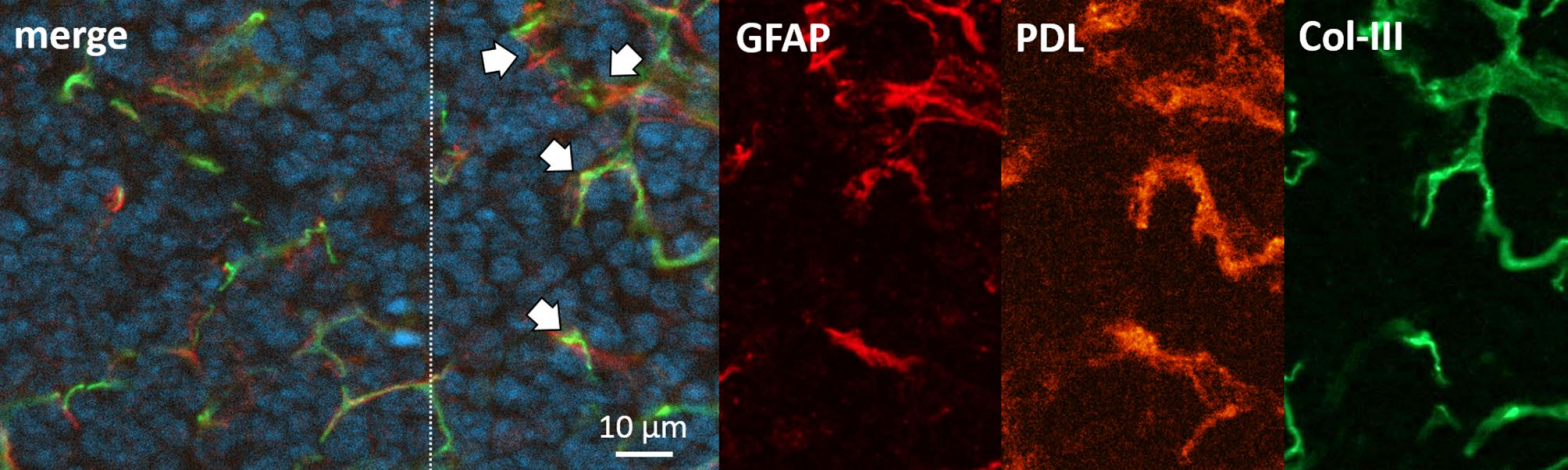
T-cell area of cervical lymph node. Immunohistochemical co-staining with polyclonal anti-GFAP antibody (DAKO) in red (directly conjugated), podoplanin (orange) and Col-III (directly conjugated, green). DAPI for nuclear staining in blue. Colocalization of GFAP, podoplanin and Col-III is abundantly found (arrows).

From the previous results, the assumption was that the GFAP-positive cells were not only Schwann cells but one or more cell types of different character. Looking more closely at the architecture and appearance of the GFAP staining, it was highly similar to the stromal cell network in the lymph node. Therefore, we performed co-staining for markers of different stromal cells. Podoplanin as a common marker for lymph node stromal cells, especially for fibroblastic reticular cells (FRC), in combination with collagen type III (Col-III) staining offered a good impression for the reticular conduit network in the lymph node and hereby the architecture of the FRC in the different areas of the lymph node (Fig. 3)^37,38^. The GFAP immunofluorescence generated by the polyclonal GFAP antibody co-stained with Col-III and podoplanin for most part of the stained tissue. Generally, these structures were highly abundant and reticularly distributed over the complete lymph node. As described above, the GFAP as well as podoplanin staining traced the typical reticular structures of the capsule, the T-cell region and also structures around the HEVs. At higher magnification we observed that GFAP and podoplanin formed double positive cell tubes in which collagen was found. This unequivocally identified the structures as FRCs (Fig. 3)*.* Nevertheless, not all GFAP-positive structures were co-localized with podoplanin. Therefore, we targeted the remaining stromal cell populations to identify GFAP-positive, podoplanin-negative cells.

**Figure 4:**
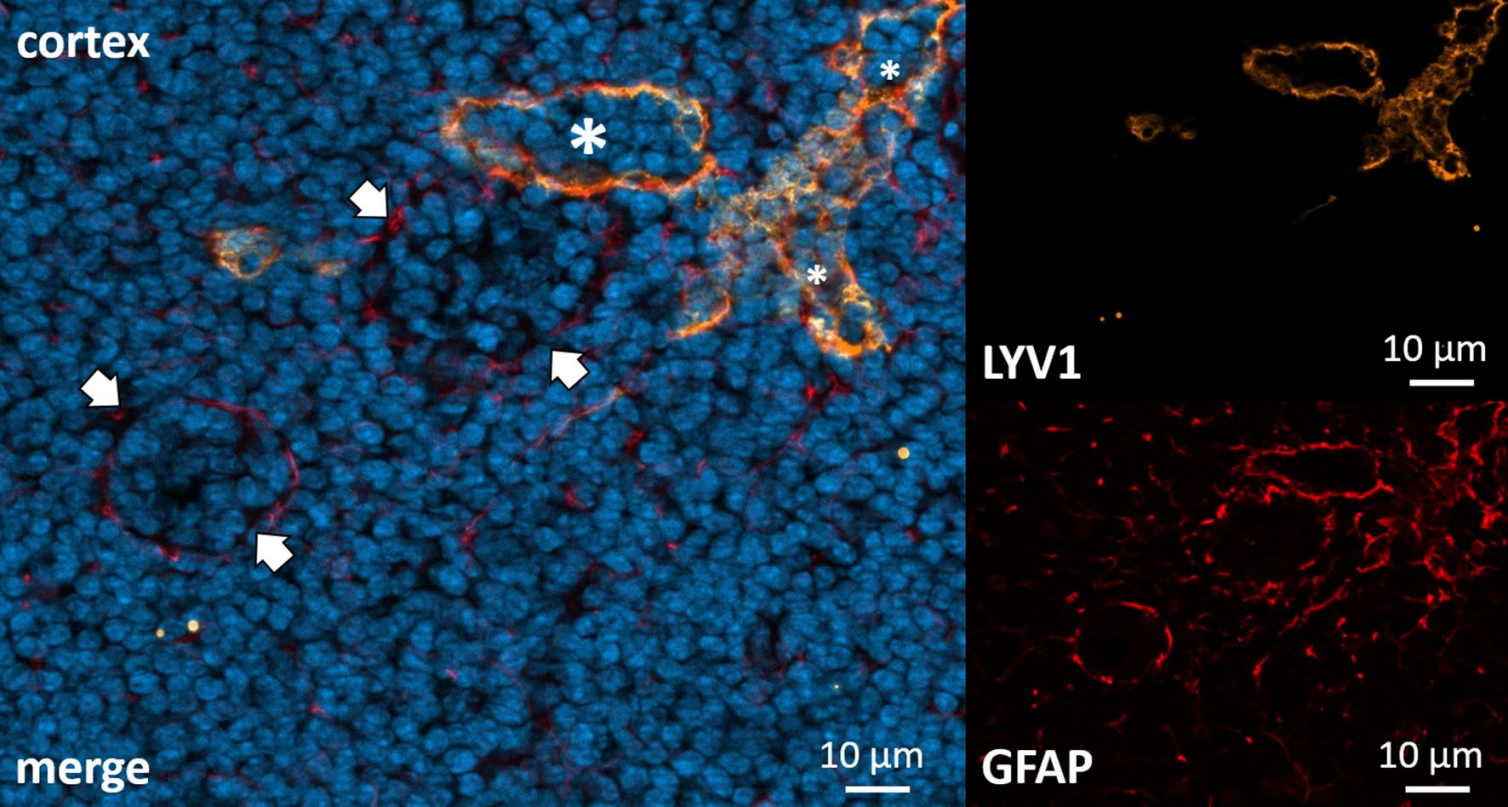
Cortex of a cervical lymph node. Immunohistochemical co-staining with polyclonal anti-GFAP antibody (DAKO) in red (directly conjugated) and anti-LYV1 in orange. DAPI for nuclear staining in blue. Lymphatic labyrinth (asterisk), here labeled by LYV1, shows colocalization with GFAP. GFAP also appears to be present in other vessel-like structures (arrows).

Lymphatic endothelial cells (LEC) are a specialized subset of endothelial cells that form lymphatic vessels and structures (lymphatic labyrinth) inside lymph nodes. LYVE-1 as a marker for LEC was stained in combination with the polyclonal GFAP antibody^3^. LYVE-1 could be detected in very close relation to GFAP (Fig. 4). LYVE-1/GFAP double positive staining marked the structures around lymphatic labyrinths (Fig. 4), in the cell layer building the floor of the subcapsular sinus as well as in the capsule.

**Figure 5:**
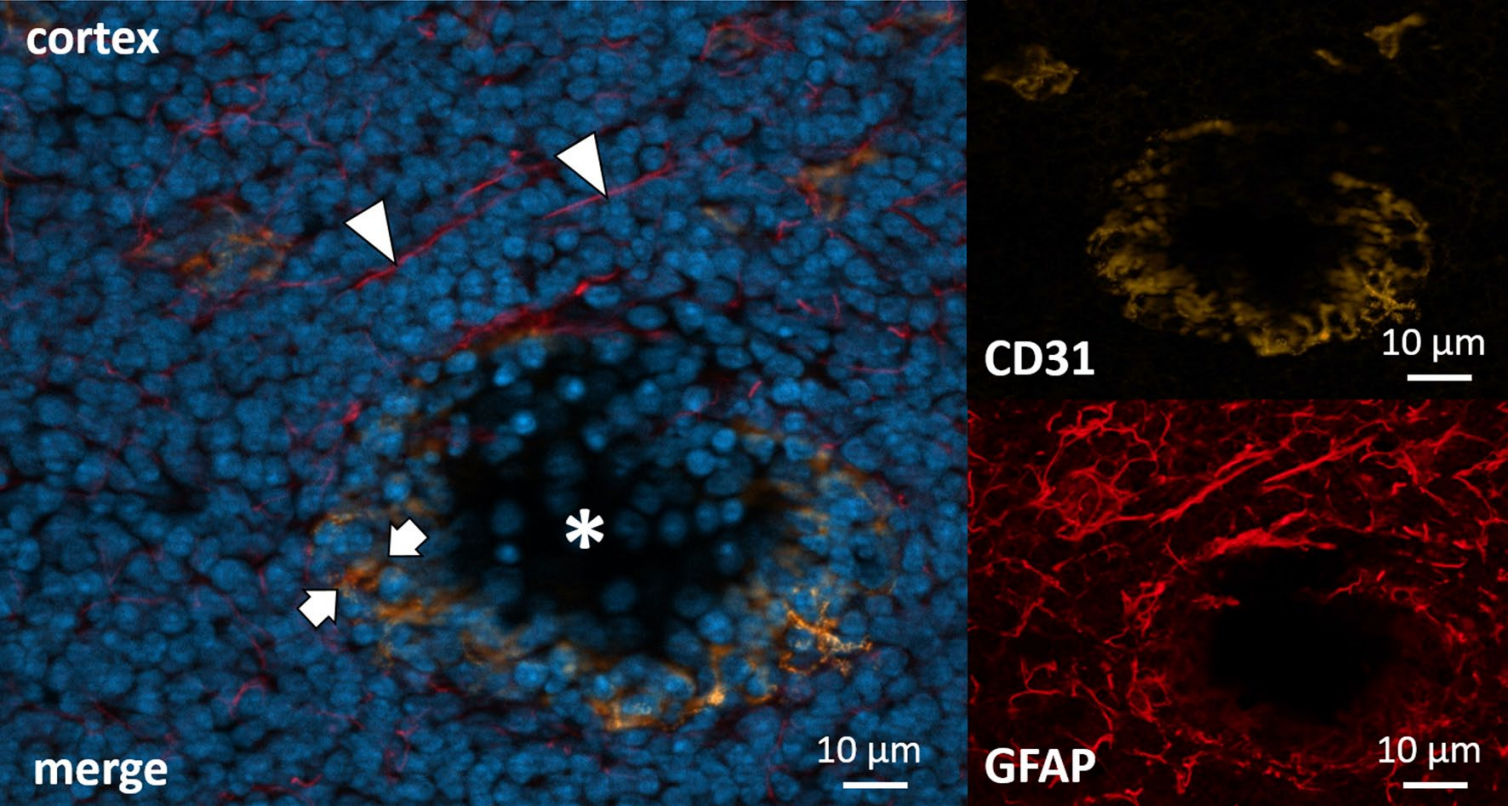
Cortex of a cervical lymph node. Immunohistochemical co-staining with polyclonal anti-GFAP antibody (DAKO) in red (directly conjugated) and anti-CD31 in orange, a marker for blood endothelium. DAPI for nuclear staining in blue. Colocalization between GFAP and CD31 is highlighted by arrows. Colocalization is clear but incomplete. GFAP immunoreactivity is also clearly visible in the parenchyma around the vessel (arrowheads), while CD31 is limited to the blood vessel (asterix).

The other major group of stromal cells, the blood endothelial cells (BEC), was stained with anti-CD31 and the polyclonal GFAP antibody. Also here, co-staining of CD31 and the polyclonal GFAP antibody was found (Fig. 5). The BECs not only lined the endothelia of arterial and venous vessels but were also located around the HEVs and clearly discriminated the blood endothelial from lymphatic endothelia in the lymph node (Fig. 5).

**Figure 6:**
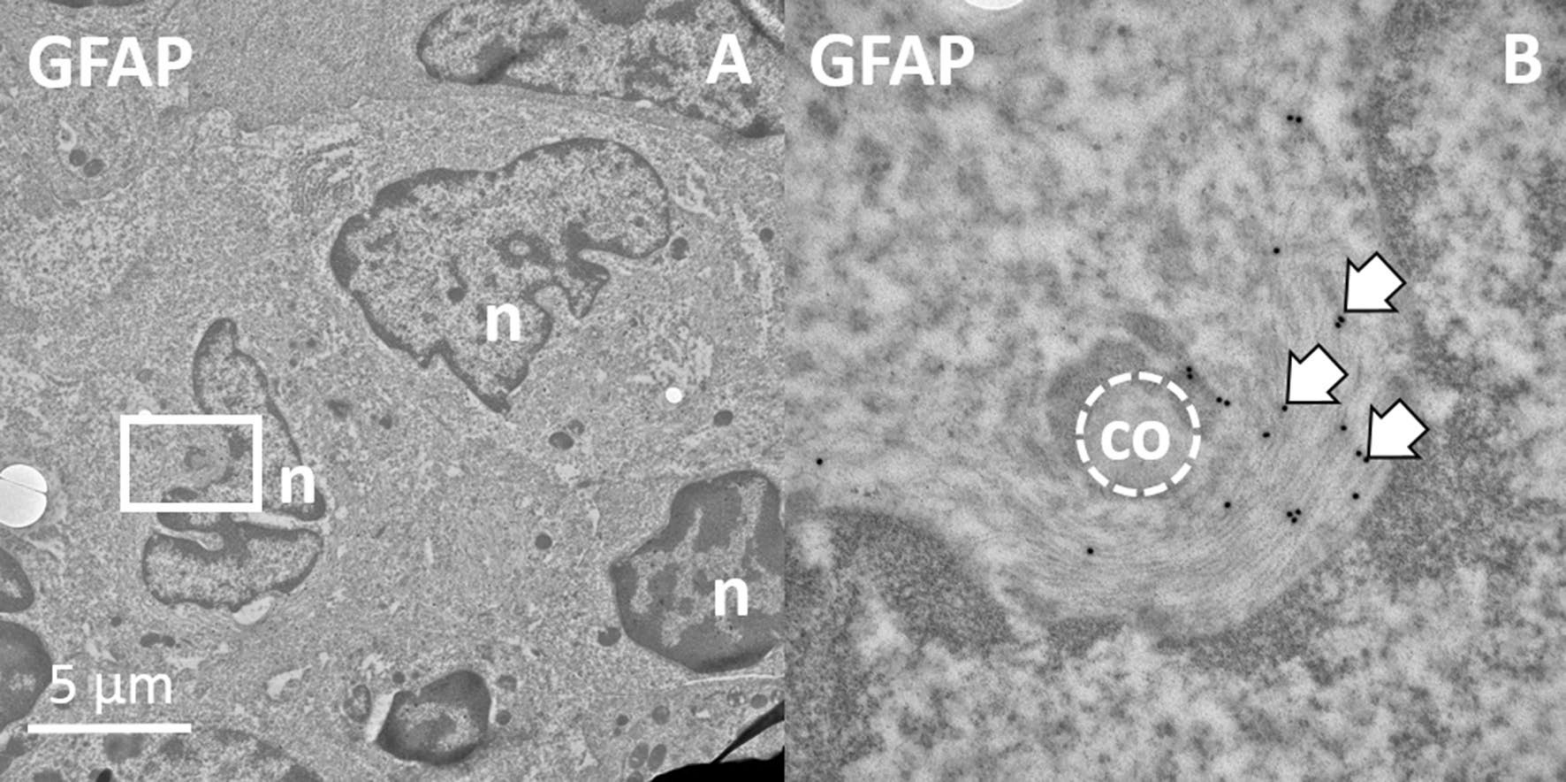
Ultrastructural image of a cell taken with a transmission emission microscope. (**A**) The picture shows several nuclei (n) with corresponding cytoplasm. (**B**) The central stromal cell (FRC) cell secretes collagen into a conduit system (co). Filamentous structures in the cytoplasm around this conduit are immune positive for GFAP (arrows) labeled using the polyclonal antibody.

The IHC clearly showed how closely GFAP immunoreactivity colocalizes with specific markers for stromal cells. And these results are independent of the used dilution and any background signals due to increased detection threshold. To gather further evidence that anti-GFAP-stained structures are an integral part of these stromal cells, we performed ultrastructural analysis using TEM and FluoroNanogold-linked secondary antibodies that were directed against the primary polyclonal GFAP antibody. As a result, we were able to depict GFAP located inside of cells whose shape and appearance matched those of stromal cells (Fig. 6A). The gold signals highlighted intermediate filaments in the cytoplasm (Fig. 6B)*.* These cells formed tunnels or tubes in which collagen was organized, leading to the assumption that they are FRCs that form the conduit system (Fig. 6).

### Detection of desmin as target intermediate filament for glial marker

**Figure 7:**
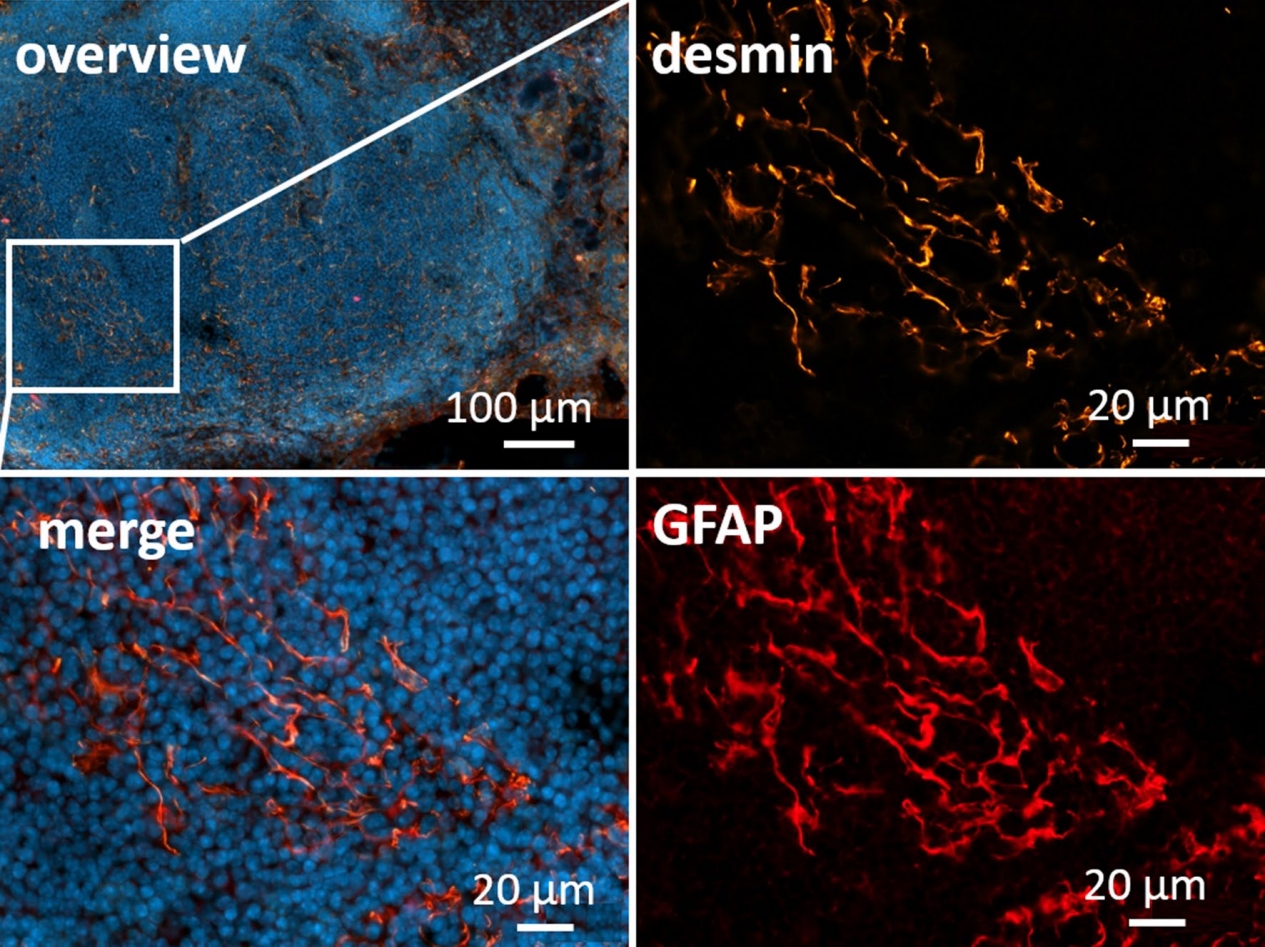
Mandibular lymph node co-stained with polyclonal anti-GFAP antibody (DAKO) in red and desmin in orange. DAPI for nuclear staining in blue. Desmin and GFAP signals are co-located throughout the tissue. The enlargement shows the reticular structures, typical for stroma cells, distributing from the cortex to the paracortex.

From the results described above, we suggested a partially unspecific binding of the polyclonal GFAP antibody to another type of intermediate filament. The different types of GFAP-positive cells that could be detected in the TEM and especially in the IHC strongly suggested apparent GFAP immunoreactivity in stromal cells. It is known that FRCs and most other stromal cells of the lymph node express desmin^4^ which is structurally related to GFAP and hence might bind GFAP antibodies. To substantiate this assumption, IHC staining was performed to test for co-localization of a specific desmin antibody and the polyclonal GFAP antibody. The majority of desmin immunoreactivity co-localized with anti-GFAP, delineating the same cellular architecture in the lymph node (Fig. 7)*.* This suggests that the polyclonal GFAP antibody detects desmin in the lymph nodes. To confirm this result, we performed two-dimensional gel electrophoresis followed by mass spectrometry analysis. This suggest that the target protein for the polyclonal GFAP antibody in the lymph node is presumably desmin (supplementary Fig. 2).

To find out if the polyclonal GFAP antibody detects only desmin exclusively in stromal cells or also glia fibrillary acid protein in lymph node glial cells, we performed additional, and again immunohistochemically staining with a monoclonal GFAP antibody.

**Figure 8:**
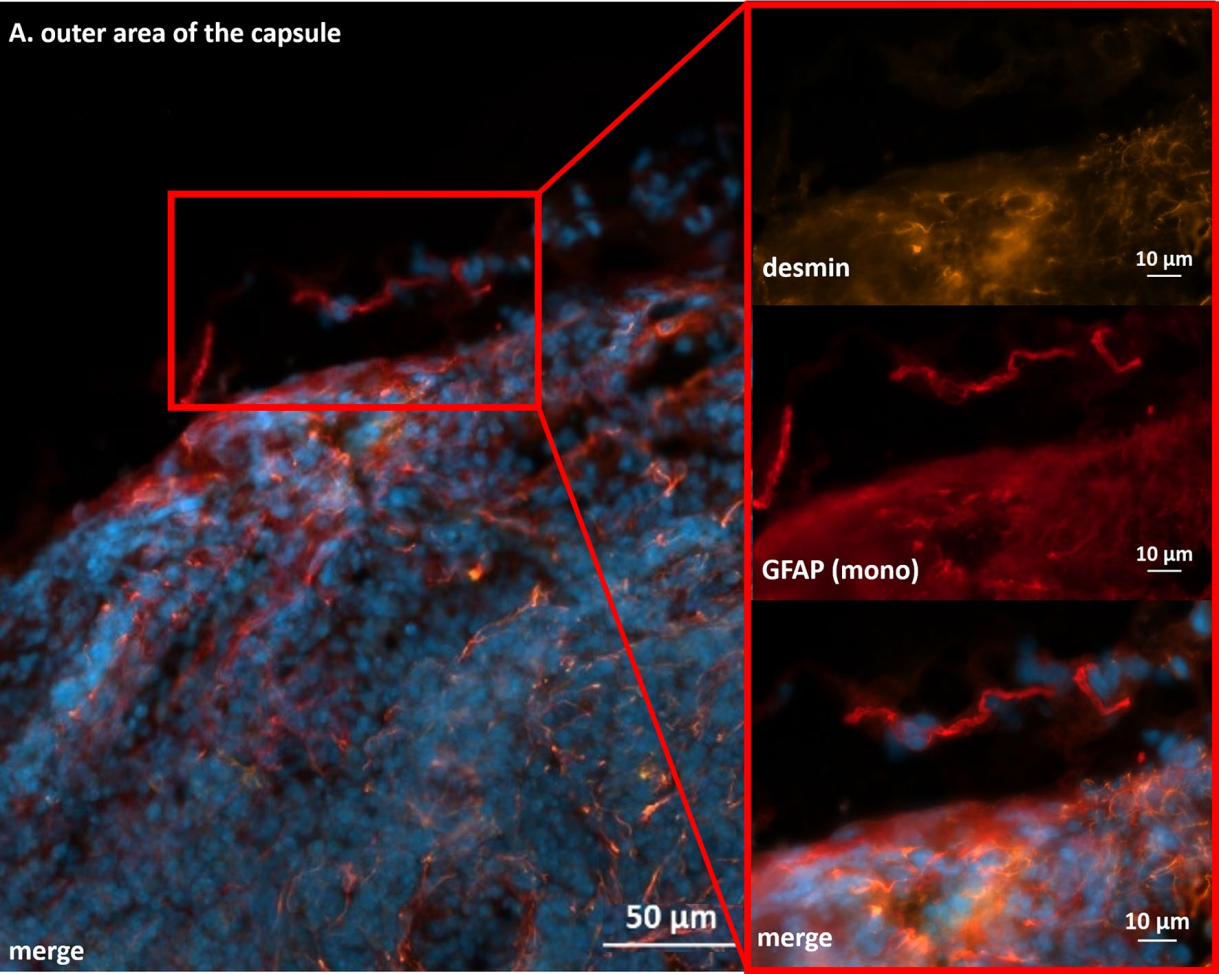
Immunhistochemical staining of cervical lymph node with monoclonal anti-GFAP antibody (red) and desmin (orange). DAPI for nuclear staining in blue. GFAP positive structures with typical morphology can be found in the outer area of the capsule of the lymph node. These structures show no staining for desmin.

**Figure 9:**
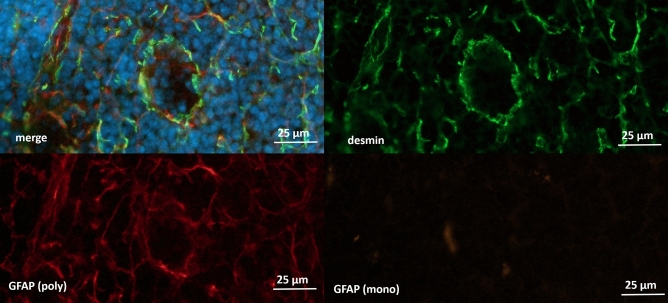
Immunhistochemical staining of cervical lymph node (cortical area) with polyclonal anti-GFAP antibody (red), monoclonal anti-GFAP antibody (orange) and desmin (green). DAPI for nuclear staining in blue. No signal with monoclonal GFAP could be found in the area of the cortex.

As expected, only a few GFAP-positive structures were found with a monoclonal antibody in the areas already described around the medulla, hilum and capsule. The labeled structures followed the shape of Schwann cells accompanying axons and could be stained with the monoclonal antibody against GFAP but not with the desmin antibody (Fig. 8). In the area of the cortex and paracortex, no IHC signals could be shown with the monoclonal antibody, whereas these areas showed strong signals for desmin and the polyclonal GFAP antibody (Fig. 9).

**Figure 10:**
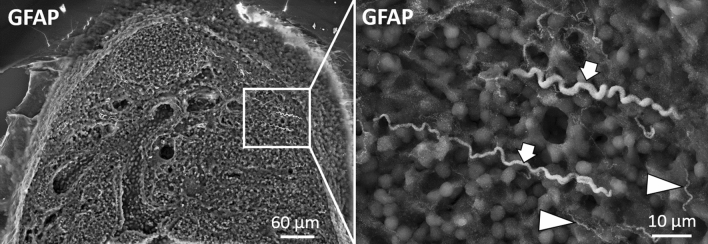
Mandibular lymph node stained with polyclonal anti-GFAP antibody (DAKO) and secondary FluoroNanogold. Two major nerves in the middle of the medullary area (see arrows in enlargement) proceeding towards the capsule and many small thin structures (arrowheads) are marked with gold.

**Figure 11:**
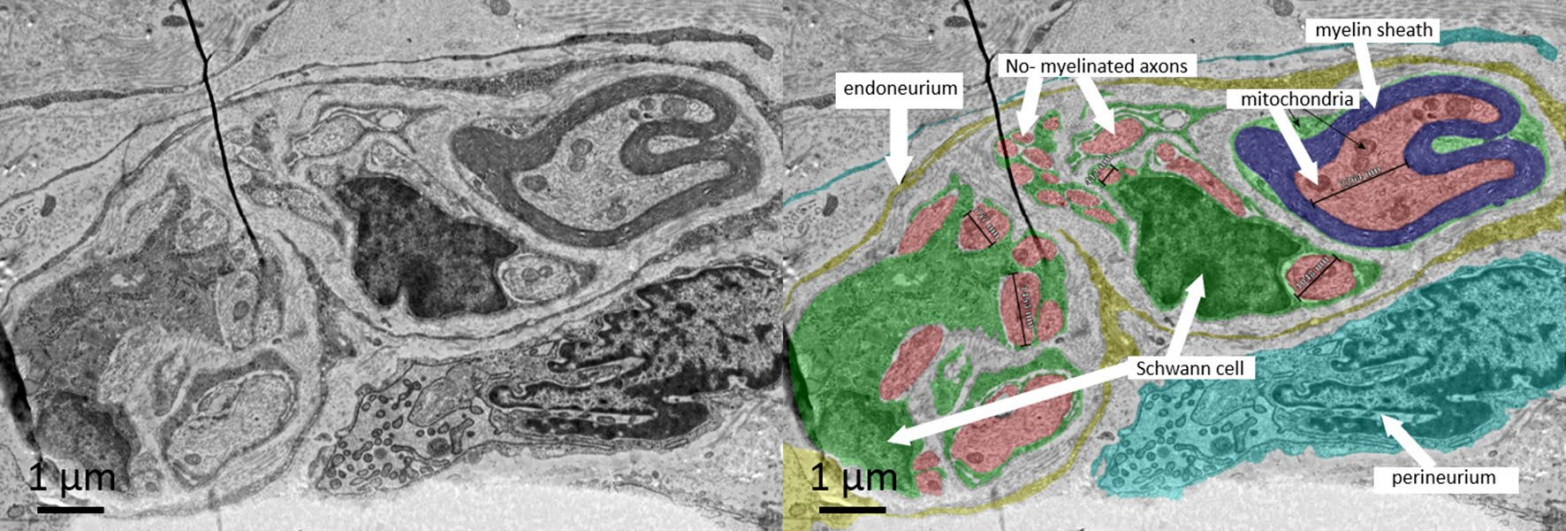
Ultrastructure image by TEM of a nerve within the medulla of an inguinal lymph node. Both, perineurium and endoneurium are well identifiable. At least two Schwann cells in this bundle accompany the non-mylelinated axon. The smallest axons have a diameter less than 400 nm. Furthermore, a myelinated axon with a diameter of ca. 2000 nm exits in this bundle.

To further characterize the GFAP-positive structures, we used ultrastructure scanning electron microscopy (SEM) analysis. We stained cryo-sections of fresh lymph nodes without chemical fixation using the polyclonal GFAP antibody combined with a secondary FluoroNanogold antibody. Structures with a twisted appearance and long directed orientation could be found in the medullary region and the hilum (Fig. 10)*.* Similar but very thin structures appeared at the capsule (Fig. 10). As observed in the IHC results, the gold-labeled structures in the cortex and paracortex seemed to be different in shape, if compared to a stromal cell network. The gold staining often traced the body of cells with a stellate shape, contacting each other at the ends of the cell processes. They formed a network within the entire T-cell area, hence resembling the typical architecture of FRCs. Also confirming the IHC results, GFAP-positive gold-labed cells targeted HEVs and surrounded them (supplementary Fig. 3). Extensive TEM analysis of the different areas in the lymph node further showed nerves with different caliber containing myelinated as well as unmyelinated axons but only in the medulla and the hilum (Fig. 11).

In summary, the polyclonal GFAP antibody detects both, desmin and GFAP, whereas the monoclonal antibody used in the present study only detects GFAP. Schwann cells, expressing GFAP as a glia-specific intermediate filament, seem to exist only in the hilum, the medulla and the capsule of the lymph nodes.

In support of this we carried out RNA fluorescence in situ hybridization (FISH) analysis against the GFAP wild type transcript. mRNA transcripts could only be detected in the areas described above and in small amounts, but always in close connection to nerves (supplementary Fig. 4).

### Unspecific binding of GFAP antibody to desmin in other organs

The fact that the polyclonal GFAP antibody recognized both, desmin and GFAP, raised the question if this antibody generally binds unspecifically to desmin, producing false positive results, or whether there is a particular desmin variant unique for the stromal cells of lymph nodes that is detected by the polyclonal GFAP antibody. To investigate this, we analyzed brain, heart, liver, thymus, spleen, skin and lung tissue from mice by Western Blot (WB) and IHC using the polyclonal GFAP antibody, a desmin antibody and the monoclonal GFAP antibody (Table 1 and supplementary Fig. 5 and 6). In the IHC staining we focused on signals similar to the desmin signal seen in the stromal cells of lymph nodes, a wide distributed filamentous structure located mainly in parenchymal areas of the organ.

**Table 1:** Summary of WB analysis and the immunohistochemistry.

	Desmin		GFAP poly		GFAP mAB	
	WB	IHC	WB	IHC	WB	IHC
Brain	–	–	X	X	X	X
Heart	X	X	–	X	–	–
Lymph node	X	X	X	X	–	–
Liver	X	X	–	X	–	X
Thymus	X	X	–	X	–	–
Spleen	X	X	–	X	–	–
Skin	X	X	–	–	–	–
Lung	X	X	–	–	–	–

Used antibodies for both applications against GFAP (polyclonal and monoclonal) and desmin. Except the brain desmin were positive in Western Blot and the IHC staining’s. Polyclonal GFAP showed signals in Western Blot in brain and lymph node and positive results in all organs excepted skin and lung using IHC. The monoclonal GFAP (GFAP mAB) antibody give signals in WB and IHC for brain tissue. IHC signals was observed in the liver.

All organs and tissues except the brain were positive for desmin in the Western Blot and the IHC staining’s. The polyclonal GFAP antibody showed positive results for brain and lymph node tissue in both approaches, Western Blot and IHC. In the other tissues, no further protein could be detected by WB using this antibody. In contrast, the IHC showed GFAP-positive structures in the heart, liver, thymus and spleen tissue. Skin and lung parenchyma tissue was negative for GFAP using this antibody.

Using the monoclonal GFAP antibody in WB analyses, GFAP protein could only be detected in brain tissue (Table 1). Using the monoclonal GFAP antibody in IHC, we could only observe GFAP-positive structures in the brain and in the liver tissue, where some nerves stained positive for GFAP. These results indicate that the polyclonal GFAP antibody produced at least partially false positive results in many other tissues and organs, in particular when used in IHC.

### Oligondendrocytes in the peripheral nervous system

**Figure 12:**
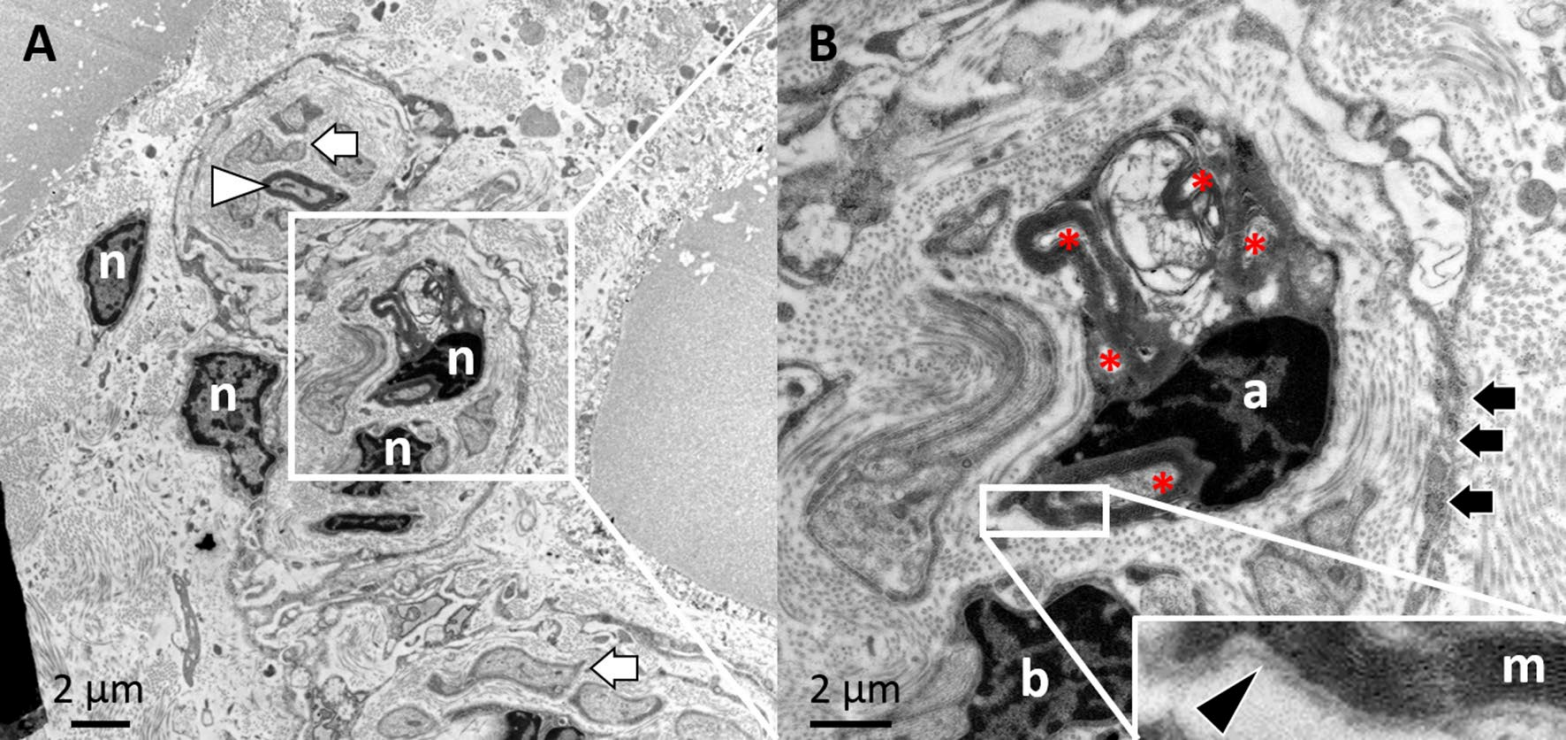
Ultrastructure image by TEM of a nerve within the medulla of an inguinal lymph node. (**A**) In addition to myelinated (white arrowhead) and non-myelinating Schwann cells (white arrow), a curios structure is found in the centrally part of the bundle. (**B**) Surrounded by the endoneurium (black arrow), there are two nerve-accompanying cells (a and b) that obviously myelinate (m) more than one axon (asterix). This structure is completely surrounded by a basement membrane (black arrowhead).

However, we made another intriguing finding in the previous TEM analysis. In the hilum and medullary area of the lymph node, we found myelinated axons, which were not individually enclosed by only one glial cell, as described for all myelinating Schwann cells in the peripheral nervous system. Here, several axons seemed to be myelinated and accompanied by just one glial cell. Up until today, this is only known from the brain with the myelinating glial cell known as oligodendrocyte (Fig. 12)*.*

**Figure 13:**
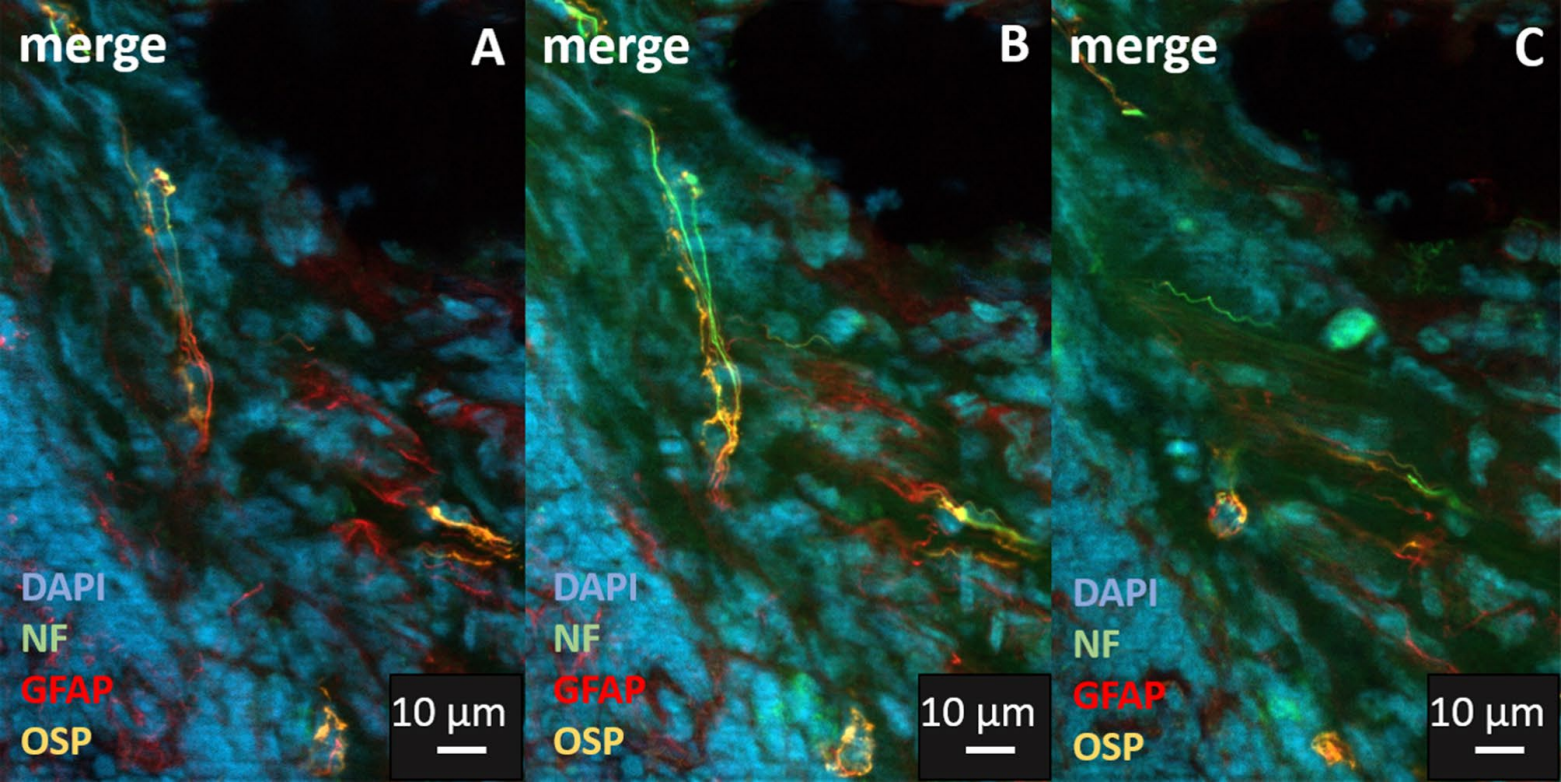
Lymph node co-stained with polyclonal anti-GFAP antibody (DAKO) in red and oligodendrocyte specific Marker (OSP) in orange, neurofilament (NF) marker in green and DAPI for nuclear staining in blue. The images from **A** to **C** show 3 different successive z levels. In image(**A**) the majority of the signal of the centrally located cell is in red (GFAP). The neurofilament signal appears to be tightly connected with the processes of the cell. This is followed in (**B**) by a comprehensive filamentous Oligo signal, which is also associated with the neurofilament. (**C**) The presumably deep cell nucleus of this cell with the surrounding double-positive signals for GFAP and Oligo.

Subsequent IHC staining with oligodendrocyte specific markers such as "oligodendrocyte-specific protein (OSP)” however, confirmed the TEM results (Fig. 13). We could observe these oligodendrocyte-like peripheral glial cells only in the hilum and medullary area of the lymph nodes. Morphologically, they appeared with a stellate shape and long cytoplasmatic extensions, resembling some of the structures that had also been positive for the monoclonal GFAP antibody in the previously described results.

## Discussion

As already known from the early 1980s and underpinned by numerous works, various efferent sympathetic fibers can be found in the lymph nodes of mice and rats, but also in other secondary lymphoid organs^36,39–41^. In addition, evidence has been found in the last two decades that suggests the presence of afferent sensory fibers in lymphoid tissues^42^. From the neural nexus which span the outer border of the lymph node to single cell innervation in the parenchyma named wAPC and wIC, our group showed in previous studies that the neural architecture in the lymph node seems to be much more complex than assumed^16,17^. With this background, the morphological picture of the innervation was to be further completed in the present work with regard to the nerve-accompanying cells, the glial cells. With the typical marker for peripheral Schwann cells, MBP, we stained discrete structures in the hilum and the medulla of the lymph node. Staining using a monoclonal antibody to GFAP, which labels GFAP but no other intermediate filaments such as desmin, was also positive in the described areas (hilum, medulla and capsule). Final confirmation of these results was then performed by fluorescence in situ hybridization (FISH); only in the areas in which long individual nerves were detected also glial cells were present. In the area of the cortex and paracortex, where we described individually innervated antigen presenting cells and other immune cells (wAPC and wIC)^17^, GFAP staining with the monoclonal antibody was absent.

Surprisingly, we also found GFAP in the putative Schwann cells besides MBP. GFAP is predominantly expressed in the CNS in astrocytes. In the periphery, GFAP is only found when regeneration or other dynamic processes occur after nerve damage^26^. This might suggests that the nerves leading to the lymph node and also the accompanying Schwann cells are in some kind of motion. 2 possibilities can be imagined here, namely that the change of the lymph node through different activity phases (immune response due to an infection event) and the concomitant increase and decrease in size requires this mechanical "flexibility". However, it would also be conceivable that the innervation in the lymph node varies independently of the volume change. Dynamic innervation of individual cells and areas of the lymph node, for example in response to an infection, could lead to an equally dynamic structure of Schwann cells^23,41,43^*.*

When compared to the monoclonal GFAP antibody, the use of the polyclonal GFAP antibody showed a completely different staining pattern. At this point we checked different dilutions of the antibody to see whether the staining pattern depends on the antibody concentration. Even with higher dilution factors, the GFAP signal can still be seen clearly. As other groups have shown in mesenteric lymph nodes, the cortex and the paracortex appeared to be regularly and massively interspersed with a network of apparently GFAP-positive cells^44^. However, this is not consistent with the observation we made with the monoclonal antibodies to MBP and GFAP that labeled only few structures in the lymph node. Also, a correlation of the staining pattern using the polyclonal GFAP antibody to the FISH data against GFAP mRNA cannot be determined. The structural arrangement of the apparently GFAP-positive cells in the T-cell area, around the HEV and also in the capsule area suggested that a co-localization with the stroma cell network exists. To analyze this, we performed an immunohistochemical counterstaining against podoplanin, CD31 and LYVE-1 which all co-localized with the GFAP-like immunoreactivity. Subsequent TEM analyzes clearly showed binding of the polyclonal GFAP antibody to intermediate filaments in stromal cells. The dominant intermediate filament in stromal cells of the lymph node is desmin^4^. This type III intermediate filament is very similar to GFAP^30^. A series of co-staining with desmin and the polyclonal GFAP antibody was performed which clearly showed that both antibodies stain the same cells, suggesting that the polyclonal antibody against GFAP cross-reacts with desmin. The overlap between these signals appeared to be almost complete, but in our opinion the immunohistochemistry was not precise enough to be really sure. To prove the cross-binding, a 2D-gel and subsequent Mass Spec analyzes were performed. The result of these analyzes shows that the polyclonal antibody against GFAP displays massively false-positive signals, at least in the lymph node. In addition to the extensive desmin-related staining in the stromal cell network, however, this antibody also detects actual GFAP, which is shown by the staining in the margins, the medulla and the hilum. Since other working groups have worked with mesenteric and therefore mucosa draining lymph nodes^44^ it should be considered, that we used skin draining lymph nodes here. But as until now there has been no description of major anatomical differences between skin and mucosa draining lymph nodes with regard to their stromal cell network, it seems most likely that the data can be compared.

The polyclonal antibody against GFAP used is one of the standard antibodies for the staining of astrocytes and other glial cells. The observations we made raise the question of how widespread the cross-reactivity between the antibody and desmin is (in any given application). To answer this question, both GFAP antibodies and the desmin antibody were used to stain various tissues and organs. The monoclonal antibody shows positive results in the brain using Western blot and immunohistochemistry, suggesting lack of cross-reactivity. The only organ that also was tested positive with the monoclonal antibody for GFAP was the liver. In addition, in all tissues and organs, excluding the brain, we detected desmin by both IHC and Western Blot. These results suggest that both the antibody against desmin and the monoclonal antibody against GFAP specifically detect desmin and GFAP, respectively.

The polyclonal antibody to GFAP, similar to the monoclonal antibody, showed positive results in the brain, both in IHC and Western blot. In the lymph nodes, there was also a positive result in the Western blot in addition to the massive IHC signals. In Western Blot analyses, no other tissue showed a signal for GFAP. However, in the IHC heart, liver, spleen and thymus were also positive. In these organs, a reticular network of apparently GFAP-positive structures was distributed over the entire tissue. It would be of major interest for subsequent work to find the reason for the inconsistent results between Western Blot and IHC with the polyclonal GFAP antibody. Further research should be done here.

But taken together, it can be concluded that the cross-reactivity between the polyclonal GFAP antibody and desmin is not limited to the lymph node, but also involves other organs and thus yields false-positive results. Hence, additional control experiments need to be performed when using this polyclonal antibody for the analysis of peripheral glial cells such as Schwann cells.

In summary, it can be said that there are no indications that glial cells are located in the parenchyma of the lymph node. Rather, the data presented here indicates that the fibers innervating the lymph nodes are only accompanied by peripheral glia until they enter the denser parenchyma of the organ. We can only guess who takes over the axon-accompanying part until their further way up to structures like the wIC or wAPC. From other areas, such as the cornea, it has been shown that stromal cells can take over the task of Schwann cellsy^45–47^. Therefore, it could be possible, that the complex population of lymph node stromal cells partially also takes over glial functions.

However, the question remains open, what happens to the incoming nerves in the medulla and how do they reach their target cells. The oligodendrocyte-like cells we found in the ultrastructure and confirmed in the IHC could be the link here. Oligodendrocytes are so far exclusively described in the CNS, where one oligodendrocyte surrounds several axons and myelinates them. To see this phenomenon in the periphery was unexpected. The myelination seems to be low here and also the axon diameter is to be regarded as low. From this it can be concluded that these are sensory fibers, presumably Aδ fibers. In our opinion, this exceptional oligo-structure could be a transition in which the sensory axon is taken from the incoming nerve and then, with the help of the processes of the oligodendrocyte, is guided into the interior of the lymph node. It would be a fascinating discovery if this cell, which is actually only present in the CNS, assumes such a role in the periphery.

## Supplementary Information

Supplementary Information.
