# Author Correction: GFAP and desmin expression in lymphatic tissues leads to difficulties in distinguishing between glial and stromal cells

**DOI:** 10.1038/s41598-021-97278-4

**Published:** 2021-08-31

**Authors:** Hauke Simon Günther, Stephan Henne, Jasmin Oehlmann, Julia Urban, Desiree Pleizier, Niclas Renevier, Christian Lohr, Clemens Wülfing

**Affiliations:** 1 Group for Interdisciplinary Neurobiology and Immunology, Biozentrum Grindel, University of Hamburg, Hamburg, Germany; 2 Division of Neurophysiology, University of Hamburg, Hamburg, Germany

Correction to: *Scientific Reports* 10.1038/s41598-021-92364-z, Published online 25 June 2021

 The original version of this Article contained an error in the spelling of the author Niclas Renevier which was incorrectly given as Nicklas Renevier. The original Article and accompanying Supplementary Information file have been corrected.

