# Supplementary material for: GFAP and desmin expression in lymphatic tissues leads to difficulties in distinguishing between glial and stromal cells
Source: Sci Rep. 2021 Jun 25;11:13322. doi: 10.1038/s41598-021-92364-z (PMC8233388; doi:10.1038/s41598-021-92364-z)
Supplement: Supplementary file 1 — Supplementary Information. [file 41598_2021_92364_MOESM1_ESM.pdf]

# Glial and stromal cells in lymphatic tissues: Caution with false binding patterns of GFAP marking desmin.

Hauke Simon Günther[1], Stephan Henne[1], Jasmin Oehlmann[1], Julia Urban[1], Desiree Pleizier[1], Niclas Renevier[1], Christian Lohr[2], Clemens Wülfing[1]

Affiliations:
1 Group for interdisciplinary neurobiology and immunology, Biozentrum Grindel, University of Hamburg
2 Division of Neurophysiology, University of Hamburg, Hamburg, Germany

**Corresponding author:**
Correspondence to: hauke@ini-research.org

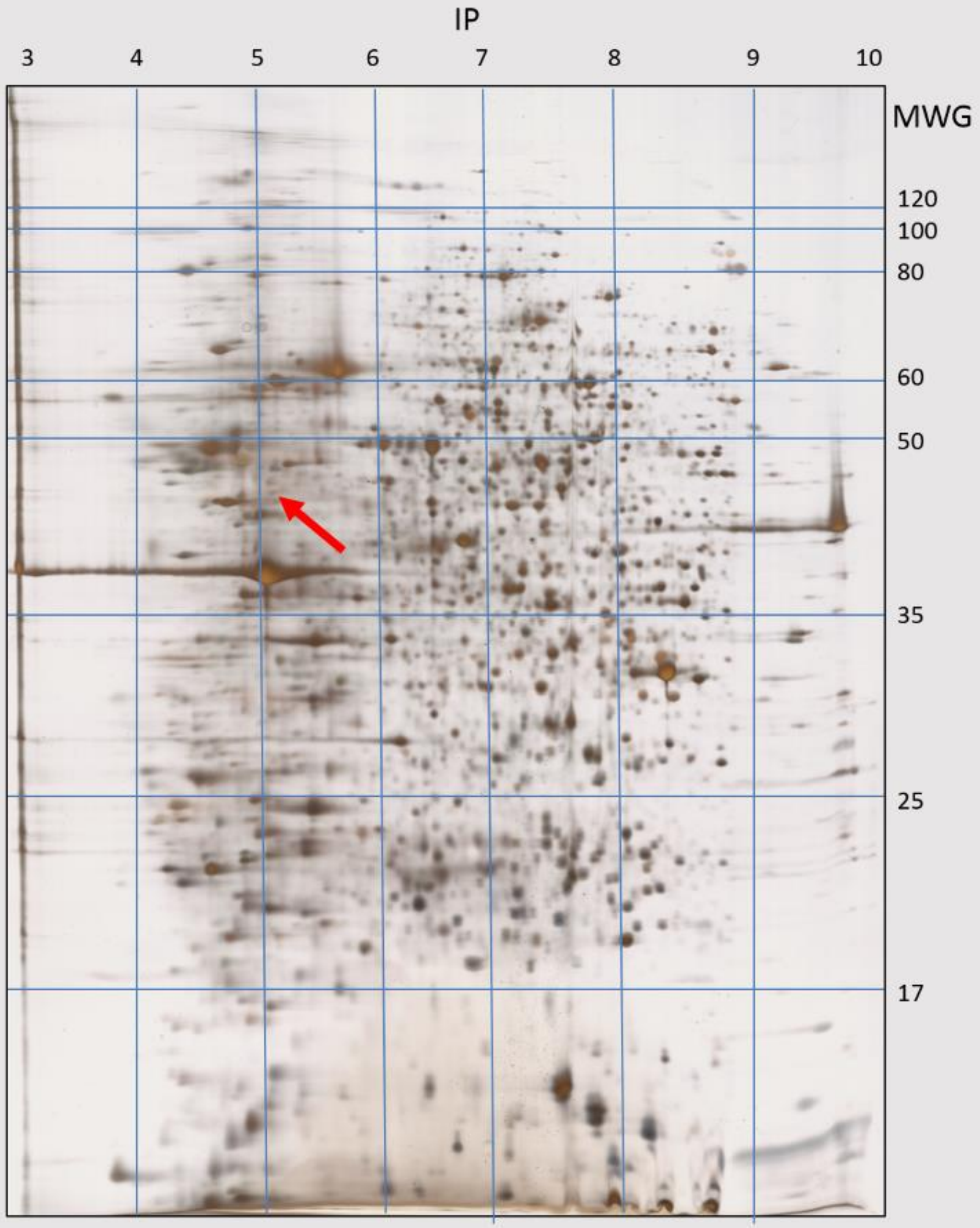


**Supplementary figure 1:** *Gel Blot of 2D-gel electrophoretic processing of pooled protein samples of lymph nodes from 2 mice (cervical lymph nodes, axillar lymph nodes, inguinal lymph nodes and popliteal lymph nodes / right and left site). Red arrow shows highly immunoreactive spot (detected by staining with polyclonal GFAP antibody (DAKO)). . Mass spectrometric analyzes identified the candidates as desmin.*

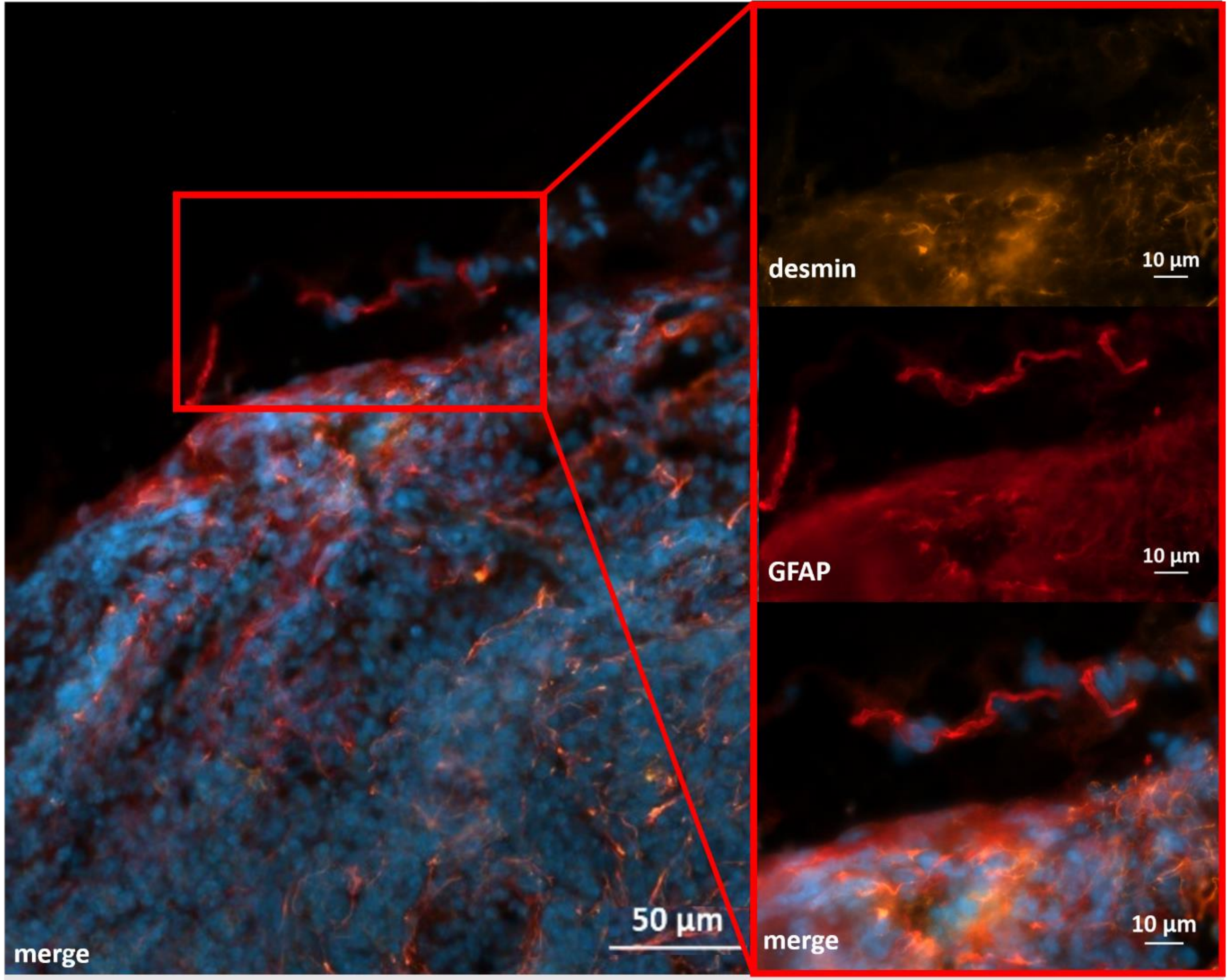


**Supplementary figure 2:** *Immunhistochemical staining of cervical lymph node with monoclonal anti-GFAP antibody (red) and desmin (orange). DAPI for nuclear staining in blue. GFAP positive structures with typical morphology can be found in the outer area of the capsule of the lymph node. These structures show no staining for desmin.*

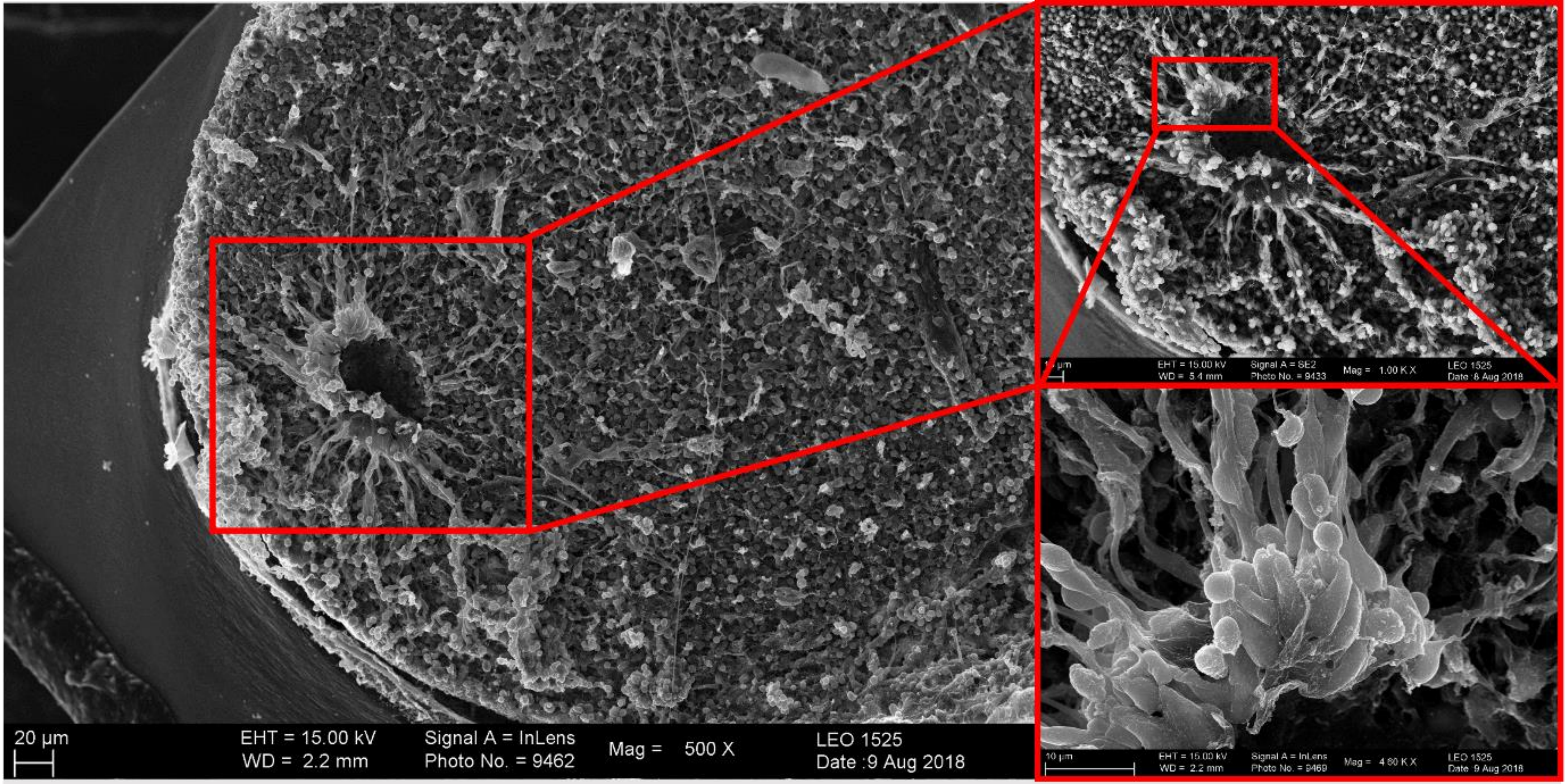


**Supplementary figure 3:** *Mandibular lymph node stained with polyclonal anti-GFAP antibody (DAKO) and* secondary FluoroNanogold™ antibody. Very thin structures were detected at the capsule as well as signals in the cortex and paracortex. They seemed to be different in shape, resembling the stromal cell network. Confirming the IHC results, cells with gold-signals targeted HEVs and surrounded them.

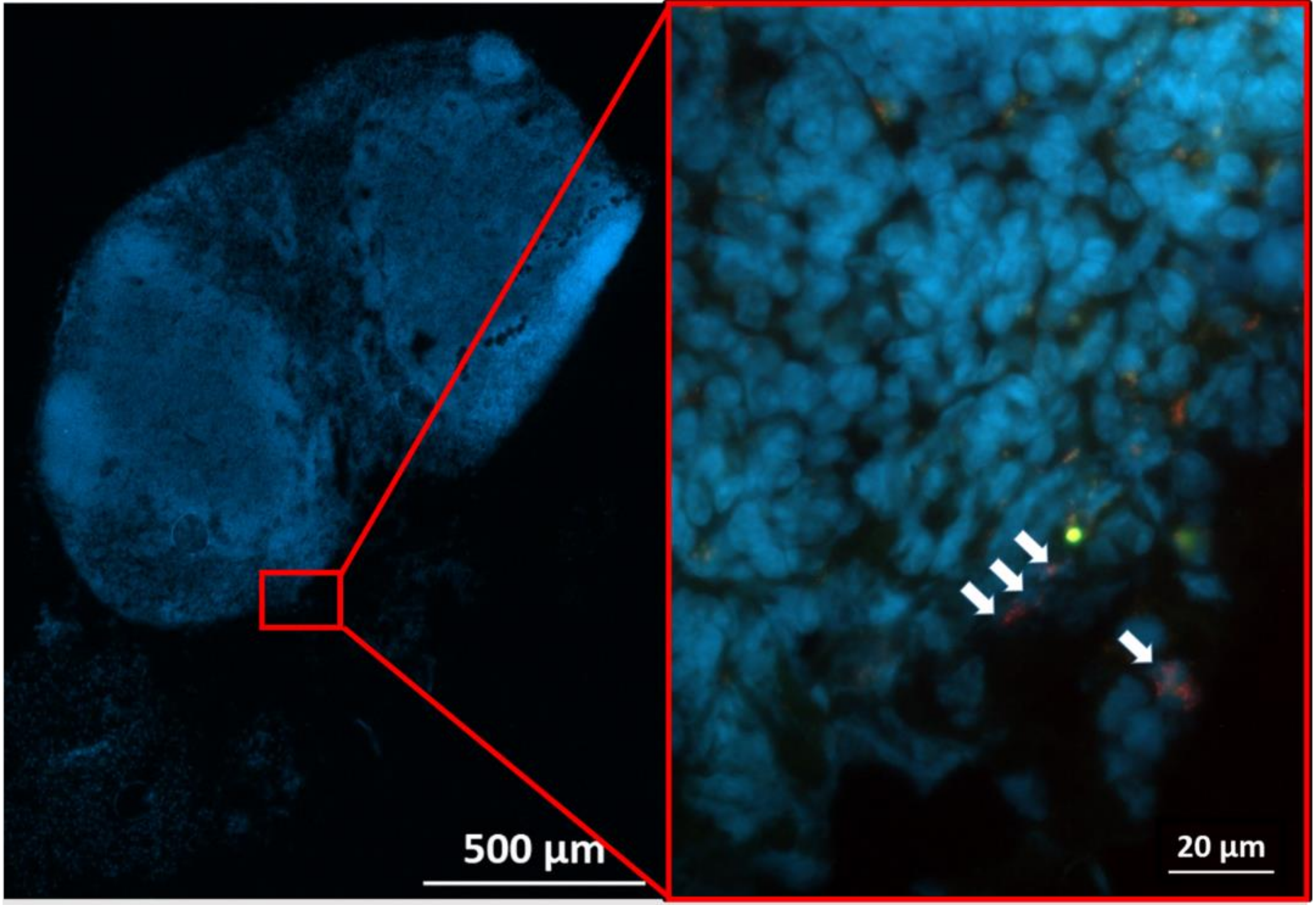


**Supplementary figure 4:** GFAP **m**RNA fluorescence in situ hybridization (FISH): mRNA transcripts were detected in the capsular. Red dots in the enlargement (right site) marked by arrows.

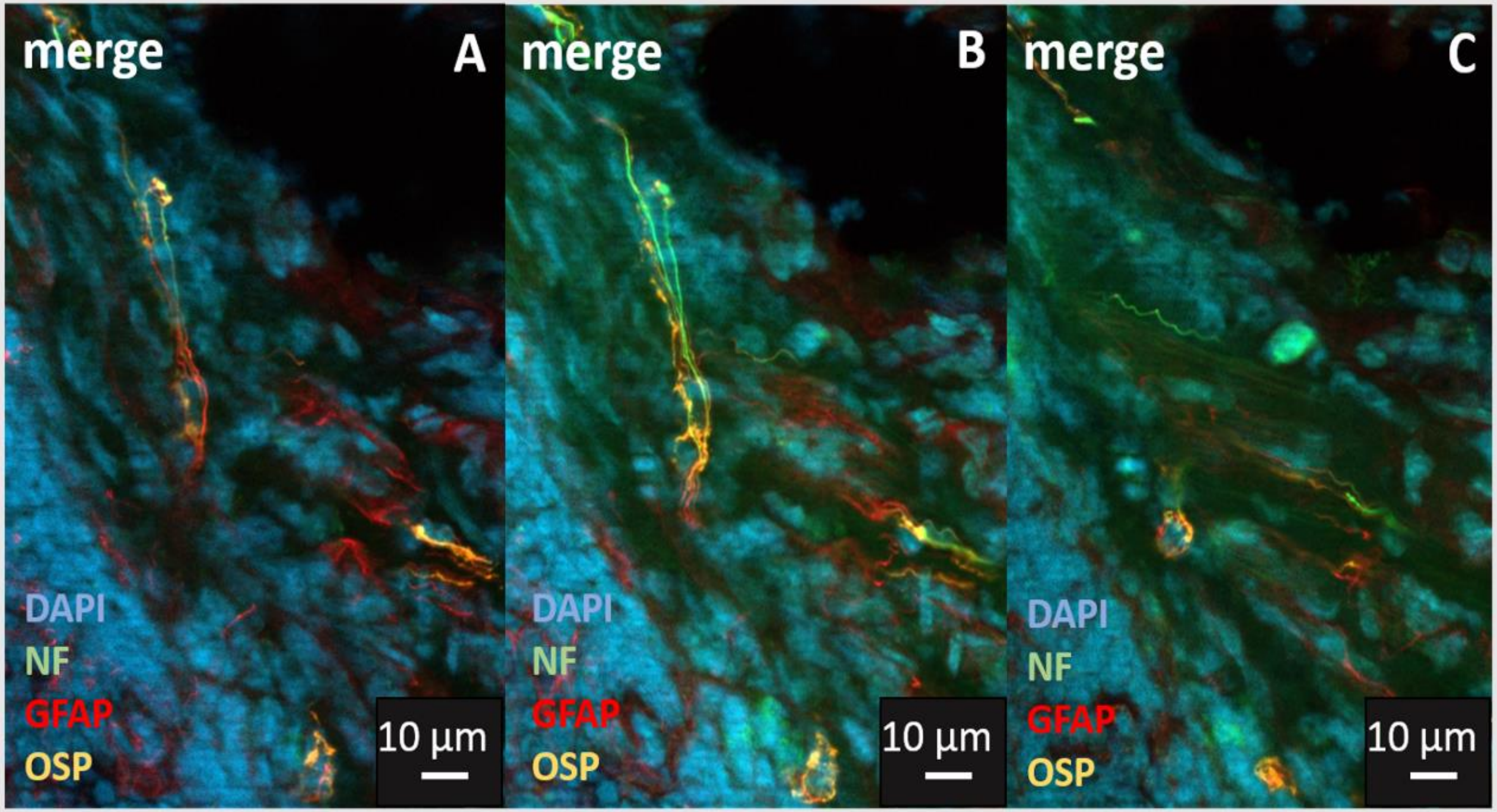


**Supplementary figure 5 :** Lymph node co-stained with polyclonal anti-GFAP antibody (DAKO) in red and oligodendrocyte specific Marker (OSP) in orange, neurofilament (NF) marker in green and DAPI for nuclear staining in blue. The images from A to C show 3 different successive z levels. In image A the majority of the signal of the centrally located cell is in red (GFAP). The neurofilament signal appears to be tightly connected with the processes of the cell. This is followed in Figure B by a comprehensive filamentous Oligo signal, which is also associated with the neurofilament. Figure C shows the presumably deep cell nucleus of this cell with the surrounding double-positive signals for GFAP and Oligo.

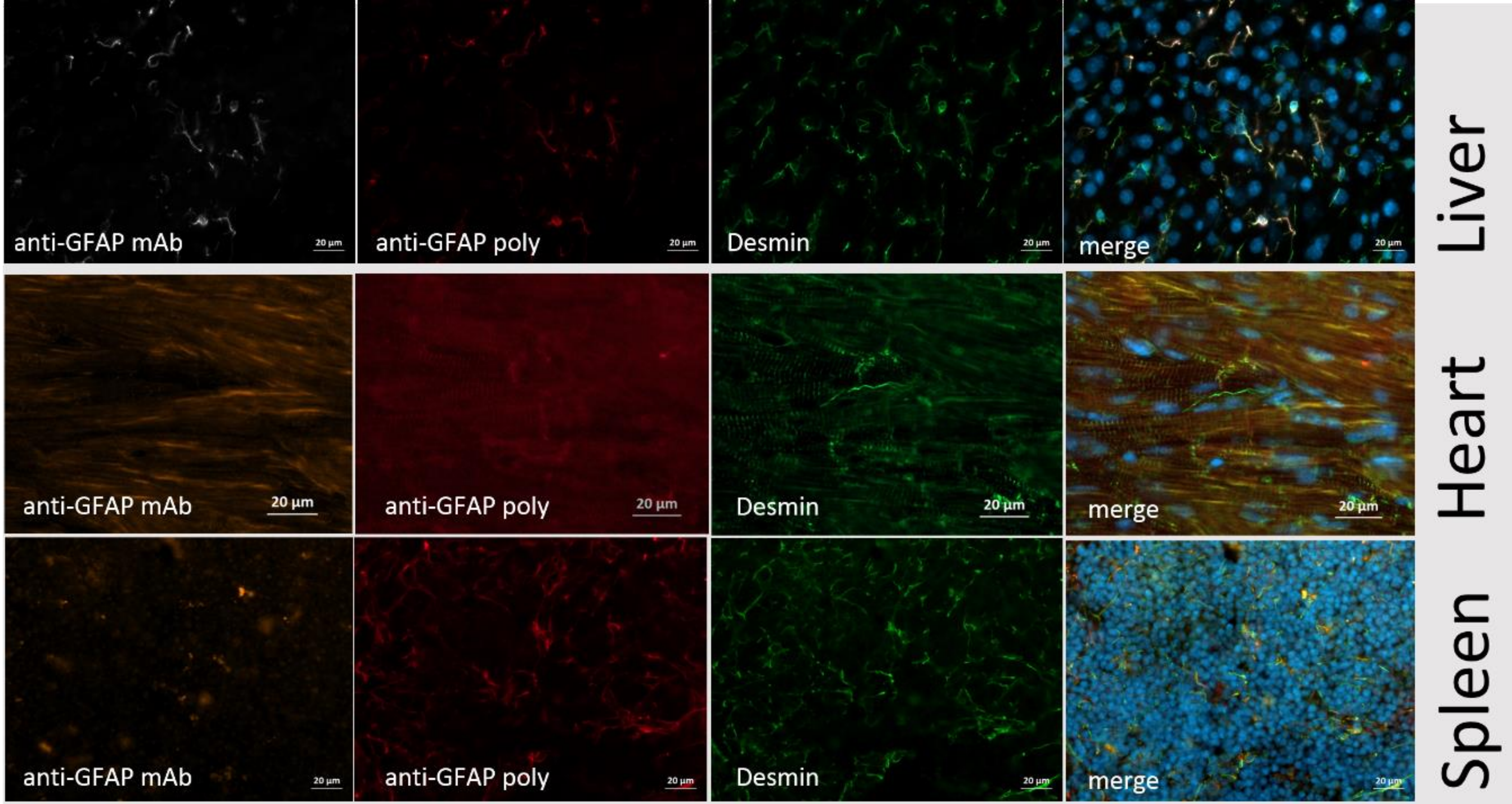


**Supplementary figure 6:** *Immunhistochemical staining of liver, heart and spleen with polyclonal anti-GFAP antibody (red), monoclonal anti-GFAP antibody (orange) and desmin (green). DAPI for nuclear staining in blue. Only liver shows triple positive signals for both GFAP and the desmin antibodies.*

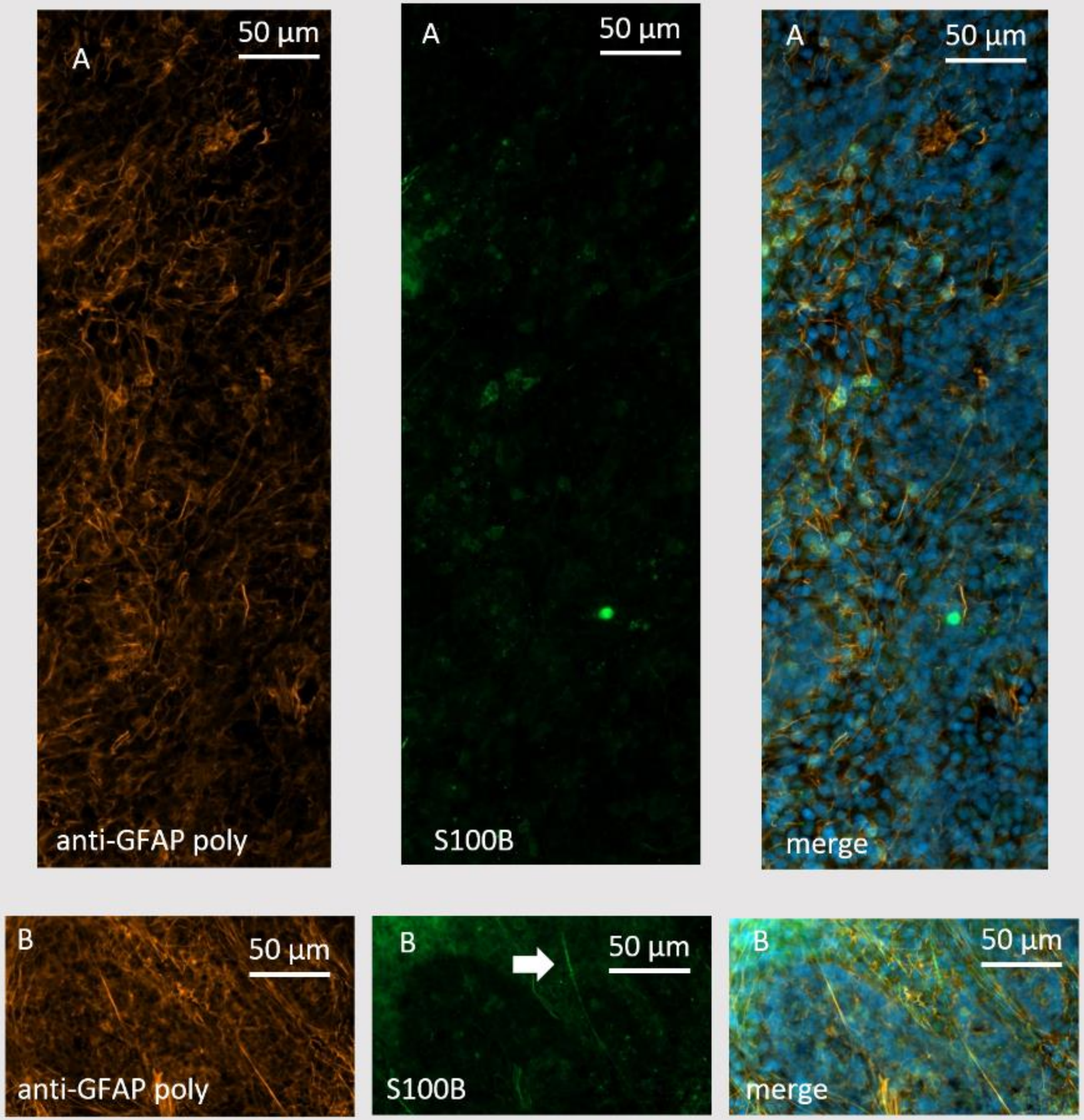


**Supplementary figure 7:**
*Immunhistochemical staining of cervical lymph node with polyclonal anti-GFAP antibody (red) and anti-S100B antibody (green). DAPI for nuclear staining in blue. No positive signals for S100B could be found in the Cortex and parenchym of the lymph node (A). Only sparse signals could be observed in the medullary region (white arrow B).*

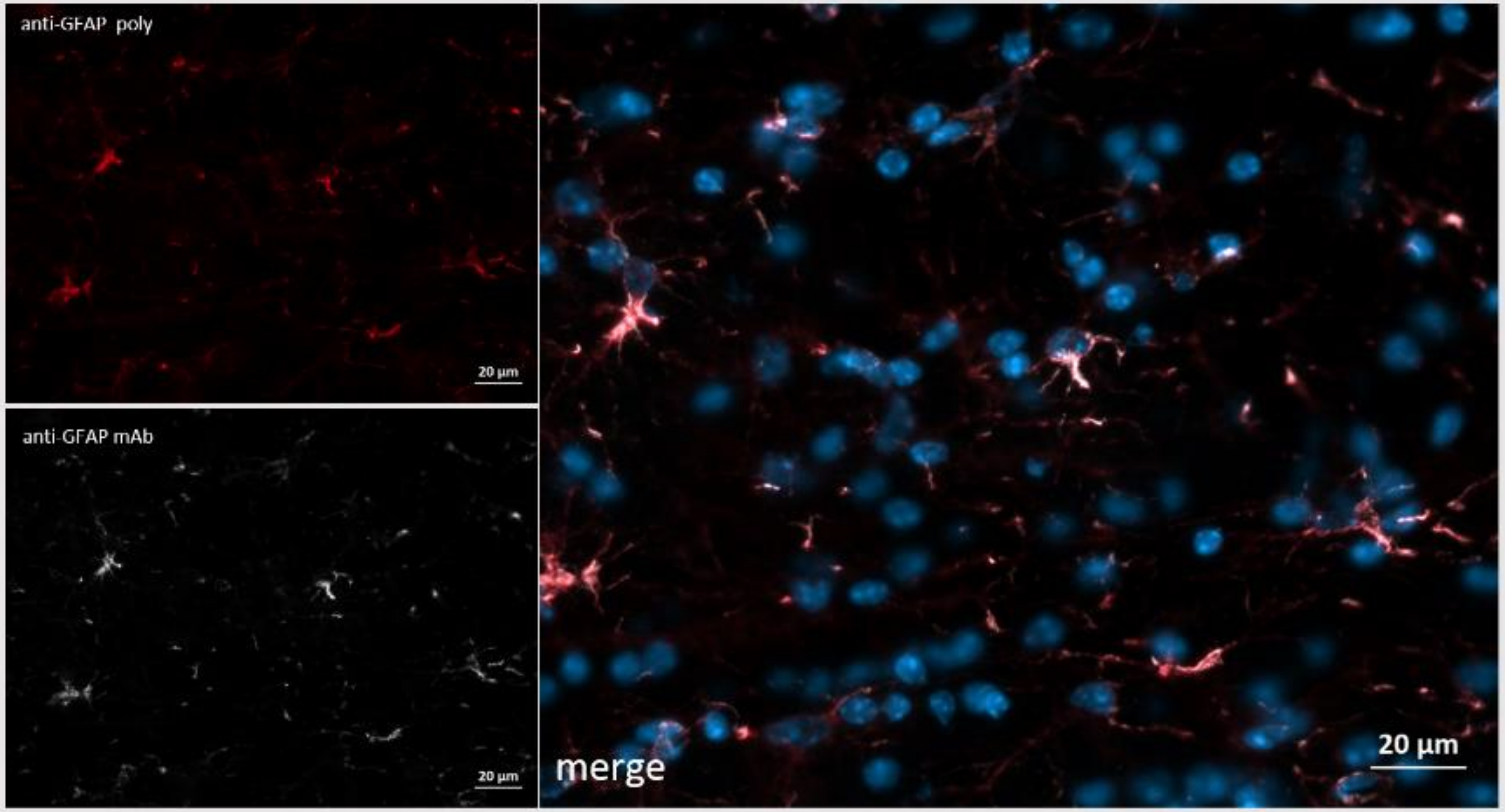


**Supplementary figure 8:** *Immunhistochemical staining of brain (mouse) with polyclonal anti-GFAP antibody (red) and monoclonal anti-GFAP antibody (white). DAPI for nuclear staining in blue. Best staining results after establishing the staining procedure confirmed 1/100 dilution factor for polyclonal GFAP and 1/500 for the monoclonal antibody.*

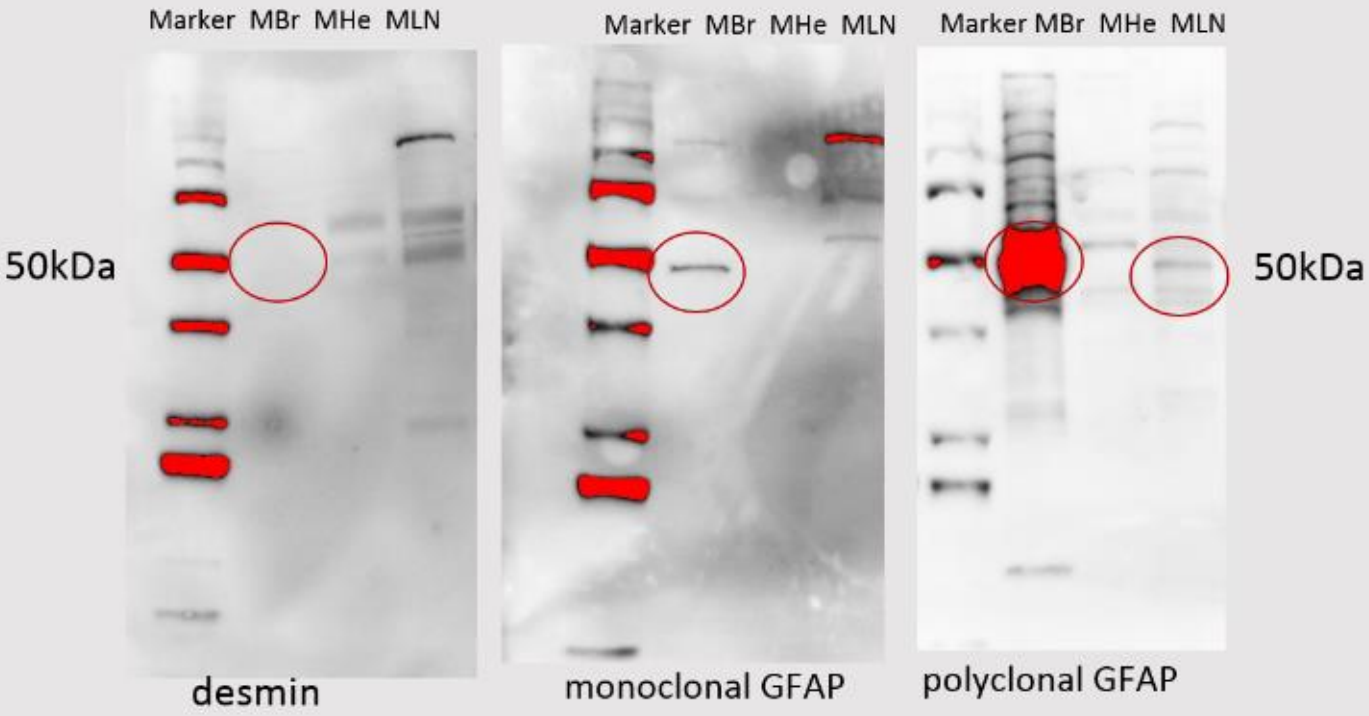


**Supplementary figure 9:** *Western Blot analysis of mouse brain (MBr), heart (MHe) and lymph nodes (MLN) with desmin, monoclonal GFAP and polyclonal GFAP antibodies. Molecular weight of GFAP approx 55kDa and desmin approx 53kDa*
